# The Pseudomonas aeruginosa Lectin LecB Causes Integrin Internalization and Inhibits Epithelial Wound Healing

**DOI:** 10.1128/mBio.03260-19

**Published:** 2020-03-10

**Authors:** Roland Thuenauer, Alessia Landi, Anne Trefzer, Silke Altmann, Sarah Wehrum, Thorsten Eierhoff, Britta Diedrich, Jörn Dengjel, Alexander Nyström, Anne Imberty, Winfried Römer

**Affiliations:** a Faculty of Biology, Albert Ludwigs University Freiburg, Freiburg, Germany; b Signalling Research Centers BIOSS and CIBSS, Albert Ludwigs University Freiburg, Freiburg, Germany; c Advanced Light and Fluorescence Microscopy Facility, Center for Structural Systems Biology (CSSB), Hamburg, Germany; d Department of Biology, University of Hamburg, Hamburg, Germany; e Department of Biology, University of Fribourg, Fribourg, Switzerland; f Department of Dermatology, Medical Center, Albert Ludwigs University Freiburg, Freiburg, Germany; g Univ. Grenoble Alpes, CNRS, CERMAV, Grenoble, France; University of California, Berkeley; Fred Hutchinson Cancer Research Center

**Keywords:** epithelial cells, fucose, glycosylation, integrins, laminin, membrane invagination, bacterial infection, glycosphingolipids

## Abstract

The opportunistic bacterium Pseudomonas aeruginosa produces the fucose-specific lectin LecB, which has been identified as a virulence factor. LecB has a tetrameric structure with four opposing binding sites and has been shown to act as a cross-linker. Here, we demonstrate that LecB strongly binds to the glycosylated moieties of β1-integrins on the basolateral plasma membrane of epithelial cells and causes rapid integrin endocytosis. Whereas internalized integrins were degraded via a lysosomal pathway, washout of LecB restored integrin cell surface localization, thus indicating a specific and direct action of LecB on integrins to bring about their endocytosis. Interestingly, LecB was able to trigger uptake of active and inactive β1-integrins and also of complete α3β1-integrin–laminin complexes. We provide a mechanistic explanation for this unique endocytic process by showing that LecB has the additional ability to recognize fucose-bearing glycosphingolipids and causes the formation of membrane invaginations on giant unilamellar vesicles. In cells, LecB recruited integrins to these invaginations by cross-linking integrins and glycosphingolipids. In epithelial wound healing assays, LecB specifically cleared integrins from the surface of cells located at the wound edge and blocked cell migration and wound healing in a dose-dependent manner. Moreover, the wild-type P. aeruginosa strain PAO1 was able to loosen cell-substrate adhesion in order to crawl underneath exposed cells, whereas knockout of LecB significantly reduced crawling events. Based on these results, we suggest that LecB has a role in disseminating bacteria along the cell-basement membrane interface.

## Abstract (precis)

Pseudomonas aeruginosa is a ubiquitous environmental bacterium that is one of the leading causes of nosocomial infections. P. aeruginosa is able to switch between planktonic, intracellular, and biofilm-based lifestyles, which allows it to evade the immune system as well as antibiotic treatment. Hence, alternatives to antibiotic treatment are urgently required to combat P. aeruginosa infections. Lectins, like the fucose-specific LecB, are promising targets, because removal of LecB resulted in decreased virulence in mouse models. Currently, several research groups are developing LecB inhibitors. However, the role of LecB in host-pathogen interactions is not well understood. The significance of our research is in identifying cellular mechanisms of how LecB facilitates P. aeruginosa infection. We introduce LecB as a new member of the list of bacterial molecules that bind integrins and show that P. aeruginosa can move forward underneath attached epithelial cells by loosening cell-basement membrane attachment in a LecB-dependent manner.

## INTRODUCTION

Pseudomonas aeruginosa is a ubiquitous Gram-negative environmental bacterium. For humans, it acts as an opportunistic pathogen and can cause severe infections, predominantly in cystic fibrosis patients (1) and immunocompromised individuals, such as HIV patients (2), patients receiving cancer treatment (3), patients with assisted ventilation (4), and patients with burn wounds (5). P. aeruginosa infections are difficult to treat because the bacterium has a high natural resistance to antibiotics and rapidly acquires new antibiotic resistances (6). In fact, several outbreaks caused by multidrug-resistant P. aeruginosa strains were recently reported (7, 8). In addition, the bacterium is able to adopt various lifestyles that allow it to evade the immune system as well as antibiotic treatment. In particular, P. aeruginosa can form biofilms (9) and invades and proliferates in host cells (10). These properties make P. aeruginosa an imminent threat for global health, and therefore, the World Health Organization (WHO) categorized P. aeruginosa as priority 1 on its recently released *WHO Priority Pathogens List for Research and Development of New Antibiotics* (11), which highlights the need to develop novel treatment strategies for P. aeruginosa infections (12).

When infecting the human body, P. aeruginosa typically encounters polarized epithelial cell layers, which function as protective barriers (10). As an opportunistic bacterium, P. aeruginosa adapts its strategy according to the circumstances that it encounters. It harnesses weak spots, for example, sites where cells divide or are extruded, to proceed to the basolateral side of epithelia (13). P. aeruginosa has also been shown to have a propensity to enter and colonize wounded epithelia (10), and there is ample experimental evidence that loss of epithelial polarity increases detrimental effects of P. aeruginosa on host cells (10). In addition, P. aeruginosa has evolved strategies to manipulate the polarity of host epithelial cells to facilitate infection (10, 14). When reaching the basolateral side, P. aeruginosa gets access to integrins, which are typically restricted to the basolateral plasma membrane of epithelial cells. Although integrins are well known as receptors for multiple pathogens (15–17), and previous studies have shown that P. aeruginosa is able to bind to α5β1-integrins in nasal epithelial cells (18) and to αvβ5-integrins in lung epithelial cells (19), the specific roles for integrins for P. aeruginosa infection remain unclear.

P. aeruginosa produces two carbohydrate-binding proteins, so-called lectins, LecA and LecB, which are also named PA-IL and PA-IIL, respectively (20). Whereas LecA is galactophilic, LecB prefers fucose (20). LecB is transported to the outer bacterial membrane, where it binds to the porin OprF, resulting in its presentation at the outer surface of P. aeruginosa (21, 22). Several lines of evidence indicate that LecB is an important virulence factor. LecB-deficient P. aeruginosa is less pathogenic (23) and shows diminished biofilm formation (21). In addition, LecB was found to abrogate ciliary beating in human airways (24) and to diminish tissue repair processes in lung epithelia (25). These findings raised the prospect of establishing alternative treatment strategies for P. aeruginosa infections by blocking LecB and stimulated ongoing efforts by several research groups to develop LecB inhibitors (26–31).

However, the functions of LecB remain difficult to pin down, because as a lectin it can bind to many different host cell receptors. Here, we demonstrate that integrins are major receptors of LecB. Moreover, we observed that LecB binding to integrins resulted in their rapid cellular uptake together with their basement membrane ligands. We provide a mechanistic explanation for this distinctive endocytosis process by showing that LecB binding to fucose-bearing lipids induces membrane invaginations and, furthermore, that LecB positions integrins in these invaginations by cross-linking integrins and lipids. As a functional consequence, purified LecB caused inhibition of cell migration and abrogation of epithelial wound healing by specifically internalizing exposed integrins in cells at the wound edge. Furthermore, we could demonstrate that the wild-type (wt) P. aeruginosa strain PAO1 is able to locally disturb cell adhesion and to crawl underneath epithelial cells. Importantly, knocking out LecB diminished the number of P. aeruginosa bacteria found underneath epithelial cells, thus implicating LecB as a virulence factor enabling bacteria to colonize host tissue along the interface between cells and the basement membrane.

## RESULTS

### Differential effects of LecB at the apical and basolateral side of polarized epithelial cells.

**FIG 1:**
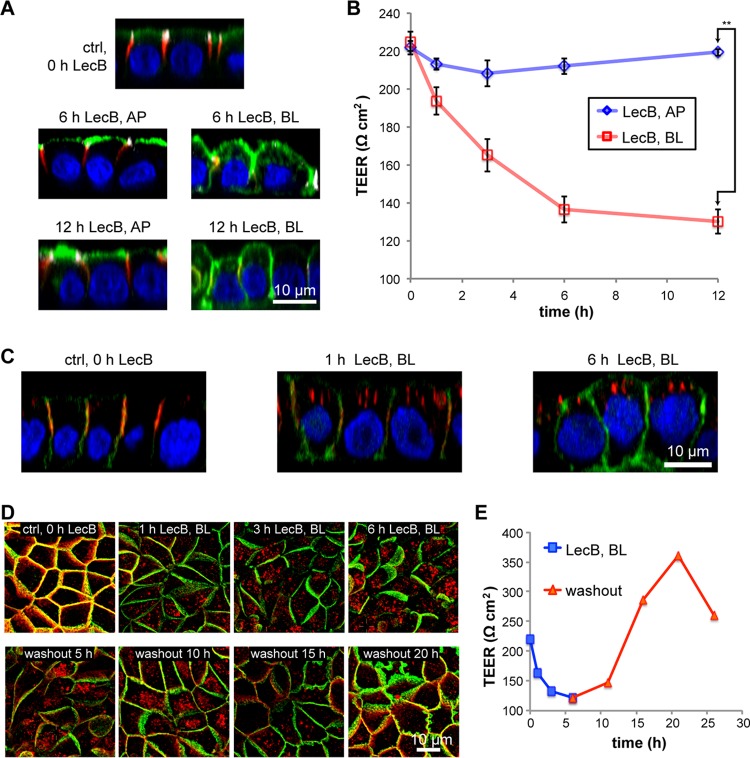
Basolateral LecB application depolarizes MDCK cells and causes integrin internalization. (A) Polarized, filter-grown MDCK cells stably expressing the apical marker GPI-GFP (green) were left untreated (ctrl) or treated apically (AP) or basolaterally (BL) with 50 μg/ml LecB for the indicated time periods, fixed, and stained with antibodies recognizing the basolateral marker β-catenin (red) and the tight junction marker ZO-1 (white); nuclei were stained with DAPI (blue). Representative sections along the apicobasal axis (*x-z* sections) extracted from confocal image stacks are shown. (B) Time course of the transepithelial electrical resistance (TEER) of MDCK monolayers treated AP or BL with LecB. The mean values from *n* = 3 experiments are displayed. For evaluating statistical significance, a paired two-sided *t* test using GraphPad Prism 5 was applied; **, *P* < 0.01. (C) LecB was applied BL to MDCK cells stably expressing PH-Akt-GFP (green) for the indicated time periods. Cells were fixed and stained for β1-integrin (red); nuclei were stained with DAPI (blue). Representative *x-z* sections extracted from confocal image stacks are depicted. (D) MDCK cells were treated with LecB as indicated, fixed, and stained for β1-integrin (red) and β-catenin (green). Maximum-intensity projections of confocal image stacks covering total cell heights are shown. (E) The time course of the TEER of MDCK cells treated BL with LecB as indicated and after washout was measured.

When P. aeruginosa infects a human body, it typically encounters first the apical pole of epithelial cells. Through induced or preexisting damages, the bacterium can access the basolateral cell pole of epithelial cells. Since the apical and basolateral plasma membranes of individual epithelial cells harbor distinct sets of membrane proteins and lipids, we investigated if LecB causes different effects when applied to the apical or basolateral side. We chose Madin-Darby canine kidney (MDCK) cells as a model system because they reliably form polarized monolayers *in vitro* (32, 33) and have been already successfully used in P. aeruginosa infection studies (14, 34). Purified LecB was able to bind apical and basolateral plasma membranes of MDCK cells (see Fig. S1A in the supplemental material). Interestingly, apical application of LecB resulted in completely different responses of the host cells than did basolateral application (Fig. 1A). After 6 h and 12 h of apical treatment with 50 μg/ml (4.3 μM) LecB, the overall morphology of the cells was intact as evidenced by staining of β-catenin (red) that remained basolateral and green fluorescent protein with a glycophosphatidylinositol anchor (GPI-GFP) (green) that remained apical. In addition, tight junction integrity was not disturbed as demonstrated by the unchanged staining of ZO-1 (white in Fig. 1A) and the preserved transepithelial electrical resistance (TEER) (Fig. 1B). In contrast, 6 h and 12 h of basolateral treatment with 50 μg/ml LecB resulted in rounded cell morphologies and severely disturbed epithelial polarity. GPI-GFP became localized all around the cells, and tight junctions almost disappeared (Fig. 1A), which was corroborated by a drastic reduction of the TEER (Fig. 1B). Importantly, these effects cannot be explained by potential LecB-mediated apoptosis or necrosis (Fig. S1B and C). Yet, the observed consequences seem to be specific for LecB, because another fucose-binding lectin, Ulex europaeus agglutinin I (UEA-I) (35), which also bound to apical and basolateral plasma membranes of MDCK cells (Fig. S1D), did not cause apparent changes in cell morphology (Fig. S1E), nor did it influence the TEER (Fig. S1F).

10.1128/mBio.03260-19.1 FIG S1 Control experiments related to Fig. 1. (A) Medium containing 50 μg/ml LecB-Alexa Fluor 488 (green) was applied to polarized filter-grown MDCK cells apically (AP) or basolaterally (BL). After fixation, nuclei were stained with DAPI (blue). Confocal *x-z* sections are displayed. (B) MDCK cells were left untreated as negative control, treated with 50 μg/ml LecB as indicated, or treated with cycloheximide (CHX; 100 μg/ml) as positive control. After cell lysis, a caspase-Glo 3/7 assay (Promega) was performed to measure the level of induced apoptosis. For evaluating statistical significance, one-way analysis of variance (ANOVA) with Tukey’s *post hoc* test using GraphPad Prism 5 was applied. ****, *P* < 0.0001; n.s., *P* > 0.05. (C) Polarized filter-grown MDCK cells were left untreated (neg. ctrl), treated AP or BL with LecB for 12 h, or treated with 0.1% Triton X-100 for 5 min (pos. ctrl.). Afterward, cells were incubated AP and BL for 5 min with 0.4% trypan blue and then washed 2 times with PBS. Since trypan blue is fluorescent upon excitation with far-red light, its presence in cells was measured using a wide-field microscope equipped with a far-red (Cy5) cube (magenta). Cells were also imaged in bright-field mode. In these images, the pores of the transwell filters are visible as small black dots. (D) Medium containing 50 μg/ml UEA-I–FITC (green) was applied to polarized filter-grown MDCK cells apically (AP) or basolaterally (BL). After fixation, nuclei were stained with DAPI (blue). Confocal *x-z* sections are displayed. (E) Polarized, filter-grown MDCK cells stably expressing the apical marker GPI-GFP (green) were left untreated (ctrl) or treated apically (AP) or basolaterally (BL) with 50 μg/ml UEA-I for the indicated time periods, fixed, and stained with antibodies recognizing the basolateral marker β-catenin (red) and the tight junction marker ZO-1 (white); nuclei were stained with DAPI (blue). Representative confocal *x-z* sections are shown. (F) MDCK cells were treated with 50 μg/ml UEA-I as indicated, and the transepithelial electrical resistance (TEER) was measured. As comparison, the data from treatment of cells with LecB from Fig. 1B are shown in gray. Download FIG S1, PDF file, 1.0 MB. Copyright © 2020 Thuenauer et al. 2020 Thuenauer et al. This content is distributed under the terms of the Creative Commons Attribution 4.0 International license.

Taken together, basolateral application of LecB dissolves epithelial polarity in MDCK cells, whereas another fucose-binding lectin, UEA-I, does not cause such effects.

### Basolaterally applied LecB binds β1-integrin and causes its internalization.

To uncover the mechanisms of LecB-induced loss of epithelial polarity, we monitored the localization of cell adhesion receptors upon basolateral LecB stimulation. This revealed a rapid and efficient internalization of β1-integrins (Fig. 1C). Interestingly, this effect was reversible after washout of LecB after 6 h (Fig. 1D), and the timing of β1-integrin internalization and return to the cell surface correlated well with decrease and increase of the TEER, respectively (Fig. 1E).

**FIG 2:**
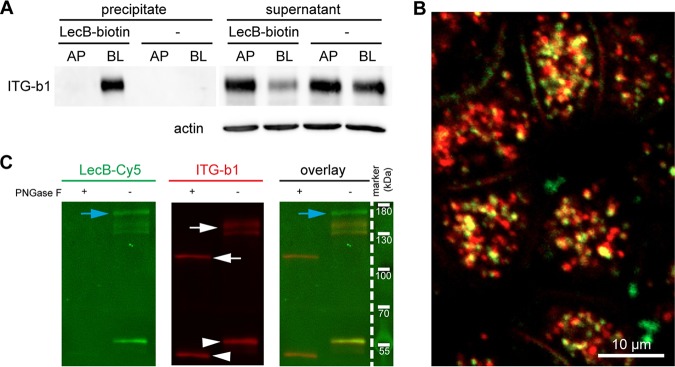
LecB directly binds to β1-integrin. (A) LecB-biotin was applied apically (AP) or basolaterally (BL) to polarized filter-grown MDCK cells, or cells were mock treated AP or BL. Cells were lysed, and LecB-biotin-receptor complexes were precipitated with streptavidin beads. Afterward, the presence of β1-integrin was probed by Western blotting in the precipitate and the remaining supernatant of the precipitation. (B) LecB-Cy3 (red) was applied basolaterally to MDCK cells for 6 h. Cells were fixed and stained for β1-integrin (green). A confocal section (*x-y* section) crossing the cells in the subapical region is displayed, since most internalized vesicles were concentrated in this region. (C) MDCK cells were lysed, and β1-integrins were immunoprecipitated and treated or left untreated with peptide-*N*-glycosidase F (PNGase F) to remove N-linked glycans. Western blot analysis of the immunoprecipitated β1-integrins was performed, and β1-integrin presence was proven by staining with anti-β1-integrin antibodies (white arrows). Also, bands from the antibody used for β1-integrin precipitation (white arrowheads) and proteins that putatively coprecipitated with β1-integrin (blue arrows) are visible. To probe the binding of LecB to β1-integrin, LecB-Cy5 was incubated with membranes (far-Western assay).

To elucidate LecB-triggered β1-integrin internalization, we first investigated if LecB binds to β1-integrin. To this end, we used LecB-biotin to precipitate LecB-receptor complexes with streptavidin beads. Western blot analysis of the precipitates showed that LecB-biotin is able to bind to β1-integrins only when applied to the basolateral side (Fig. 2A). The binding of LecB-biotin to β1-integrin appeared to be strong, since LecB-biotin was able to extract approximately 75% of total β1-integrin when applied to the basolateral side, as quantified from the band intensities of the Western blot. In addition, fluorescently labeled LecB colocalized with internalized β1-integrins (Fig. 2B) and was able to bind immunoprecipitated β1-integrin in a far-Western assay depending on the β1-integrin glycosylation status (Fig. 2C), which provides complementary evidence for the capacity of LecB to bind to glycosylated β1-integrin.

In summary, LecB recognizes β1-integrin at the basolateral cell surface and causes its rapid internalization.

### α3-Integrin and laminin are also internalized and degraded upon basolateral LecB application.

**FIG 3:**
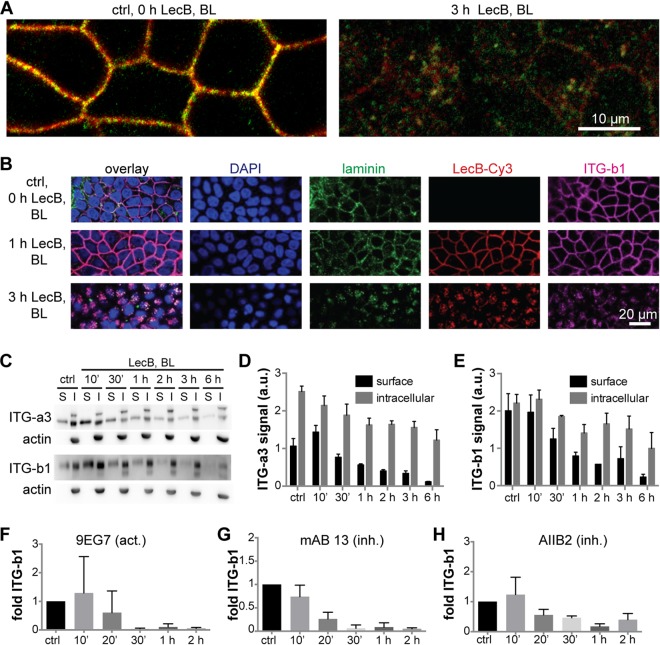
LecB internalizes α3β1-integrin regardless of its activation status and together with laminins. (A) MDCK cells were treated with LecB as indicated, fixed with methanol, and stained for α3-integrin (red) and β1-integrin (green). Confocal sections (*x-y* sections) through the middle of the cells extracted from confocal image stacks are shown. (B) MDCK cells were treated with LecB-Cy3 (red) as indicated, fixed, and stained for panlaminin (green) and β1-integrin (ITG-b1; magenta); nuclei were stained with DAPI (blue); *x-y* confocal sections through the middle of the cells are depicted. (C to E) MDCK cells were treated with LecB as indicated, and surface biotinylation from the basolateral side was performed. After precipitation of biotinylated proteins, the precipitates representing the surface fraction (S) and the supernatant representing the intracellular fraction (I) were subjected to Western blot analysis, and α3-integrins and β1-integrins were probed, as well as actin to control for purity of the surface fractions. Quantification for α3 (D) and β1 (E) subunit-composed integrins from *n* = 3 independent experiments. (F to H) LecB was applied basolaterally to MDCK cells for the indicated time periods followed by basolateral application of activation-specific anti-β1-integrin antibodies to live cells. After fixation, the signal from bound anti-β1-integrin antibodies in randomly chosen regions of interest was measured and normalized to the cell number in the regions (*n* = 5 for one experiment). The graphs show the mean value from *n* = 3 experiments with the activating anti-β1-integrin antibody 9EG7 (F) and the inhibitory anti-β1-integrin antibodies monoclonal antibody (MAb) 13 (G) and AIIB2 (H).

From the far-Western assay in Fig. 2C, it can be seen that LecB recognized not only glycosylated β1-integrin but also other receptors that were presumably coprecipitated during β1-integrin immunoprecipitation (blue arrows). Hence, in a next step, we identified basolateral interaction partners of LecB by LecB-biotin coprecipitation followed by mass spectrometry (MS) analysis and found 65 profoundly enriched proteins (Table S1). This analysis revealed that LecB is able to pull down virtually all integrins expressed by MDCK cells (36), and also, many proteins known to interact with integrins, such as tetraspanins, basigin, and epidermal growth factor receptor (EGFR), were detected. From this, it appears that integrins are major cellular receptors of LecB. We focused our further analysis on α3β1-integrin and were able to demonstrate that α3-integrins are cointernalized with β1-integrins upon basolateral LecB application (Fig. 3A). Surprisingly, also the major ligands of α3β1-integrin expressed by MDCK cells, laminin-332 and/or -511 (37), were cointernalized (Fig. 3B). This suggests that LecB is able to cause endocytosis of intact α3β1-integrin–laminin complexes. To measure the dynamics of α3β1-integrin internalization, we carried out surface biotinylation experiments (38). These experiments confirmed the rapid LecB-triggered internalization of α3- and β1-integrin subunits (Fig. 3C to E). In addition, the surface biotinylation experiments revealed that also the intracellular amount of α3- and β1-integrin subunits decreased upon LecB stimulation, suggesting a degradation of internalized integrins. Consistently, LecB-mediated reduction of integrins was also detected when whole-cell lysates were subjected to Western blot analysis (Fig. S2A). Loss of integrins by degradation is further supported by our finding that internalized integrins after basolateral LecB treatment showed a time-dependent increase in colocalization with the late endosome marker Rab9 (Fig. S2B and C) and the lysosome marker Lamp1 (Fig. S2D and E).

10.1128/mBio.03260-19.2 FIG S2 Control experiments related to Fig. 3. (A) The total amounts of β1-integrin were probed by Western blotting (WB) in MDCK cells basolaterally treated with LecB. (B and C) MDCK cells were basolaterally treated with LecB as indicated, fixed, and stained for Rab9 (green) and β1-integrin (red), and nuclei were stained with DAPI (blue). (B) Representative confocal sections (*x-y* sections) through the middle of the cells. (C) Quantification of the Manders overlap-coefficient between Rab9 and β1-integrin from *n* = 3 independent experiments. (D and E) MDCK cells were basolaterally treated with LecB as indicated, fixed, and stained for Lamp1 (green) and β1-integrin (red), and nuclei were stained with DAPI (blue). (D) Representative confocal sections (*x-y* sections) through the middle of the cells. (E) Quantification of the Manders overlap-coefficient between Lamp1 and β1-integrin from *n* = 3 independent experiments. (F) LecB was applied basolaterally to polarized filter-grown MDCK cells for the indicated time periods followed by basolateral application of activation-specific anti-β1-integrin antibodies (9EG7, MAb 13, and AIIB2) to live cells. After fixation, the signal from bound anti-β1-integrin antibodies was measured with a confocal microscope. Representative maximum-intensity projections of confocal image stacks covering full cell heights are displayed. Download FIG S2, PDF file, 2.1 MB. Copyright © 2020 Thuenauer et al. 2020 Thuenauer et al. This content is distributed under the terms of the Creative Commons Attribution 4.0 International license.

10.1128/mBio.03260-19.8 TABLE S1 List of basolateral LecB interaction partners identified by SILAC MS. Download Table S1, PDF file, 0.2 MB. Copyright © 2020 Thuenauer et al. 2020 Thuenauer et al. This content is distributed under the terms of the Creative Commons Attribution 4.0 International license.

In the surface biotinylation experiments, we were not able to distinguish between active and inactive β1-integrins. Thus, we devised an alternative strategy in which we applied activation-specific β1-integrin antibodies to the basolateral surface of live cells. This approach revealed that LecB internalizes active and inactive β1-integrins at similar kinetics (Fig. 3F to H and Fig. S3F), which indicates that the activation status of β1-integrins does not play an important role in LecB-mediated integrin internalization.

**FIG 4:**
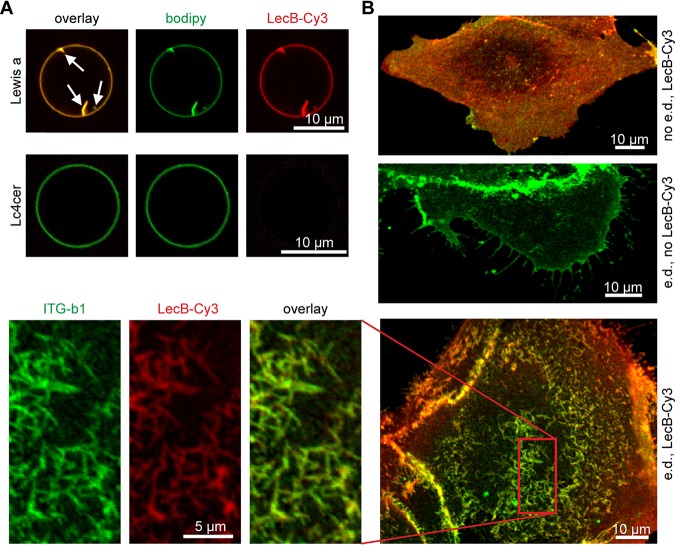
Mechanism of LecB-mediated integrin internalization via cross-linking glycosphingolipids and integrins. (A) LecB-Cy3 (15 μg/ml, red) was applied to GUVs containing fucosylated glycosphingolipids bearing the Lewis a antigen (Lewis a) or the nonfucosylated precursor lactotetraosylceramide (Lc4cer) and BODIPY FL C_5_ HPC (bodipy; green) as a membrane marker. Confocal sections along equatorial planes of the GUVs are displayed; arrows point to membrane invaginations caused by LecB. (B) Subconfluent MDCK cells grown on glass coverslips were energy depleted (e.d.) or left untreated (no e.d.). LecB-Cy3 (red) was applied to the cells for 1 h, and cells were fixed and stained for β1-integrin (green). Confocal *x-y* sections at the level of the cell adhesion to the glass coverslip are displayed.

10.1128/mBio.03260-19.3 FIG S3 Control experiments related to Fig. 4, part 1. LecB-Cy3 (15 μg/ml, red) was applied to GUVs containing β-BODIPY FL C_5_-HPC (bodipy; green) as a membrane marker and glycosphingolipids bearing antigens from the type 1 series (lactotetraosylceramide [Lc4cer; nonfucosylated precursor as negative control], H type 1, A type 1, B type 1, A Lewis b, Lewis a, or Lewis b) or the Lewis X antigen from the type 2 series. Schematic structures of the glycolipids are also depicted on the left. Confocal sections along equatorial planes of representative GUVs are displayed. Download FIG S3, PDF file, 0.8 MB. Copyright © 2020 Thuenauer et al. 2020 Thuenauer et al. This content is distributed under the terms of the Creative Commons Attribution 4.0 International license.

Taken together, LecB binds integrins, including α3β1-integrin, and causes their internalization and degradation regardless of their activation status and bound basement membrane ligands.

### Membrane invagination by LecB and LecB-mediated cross-linking of fucosylated lipids with β1-integrin can explain LecB-triggered integrin internalization.

Endogenous lectins, like galectin-3, were previously shown to be able to mediate integrin internalization (39, 40). The proposed mechanism for galectin-3-mediated integrin internalization is that galectin-3 is able to cause plasma membrane invaginations by binding to glycolipids and also drags integrins into the invaginated membrane regions by functioning as a cross-linker between glycolipids and integrins.

Since LecB is a tetramer with four opposing fucose-binding sites (41), which represents an ideal geometry for a potential cross-linker, we investigated if a galectin-like mechanism could explain LecB-mediated β1-integrin internalization.

In a first step, we examined if binding of LecB to fucosylated glycosphingolipids is sufficient to induce membrane invaginations in giant unilamellar vesicles (GUVs) (42). Indeed, GUVs containing glycosphingolipids that bear the fucosylated Lewis a antigen (Fig. 4A) or other fucosylated glycosphingolipids (Fig. S3) showed invaginations immediately after LecB application, whereas control GUVs with the nonfucosylated glycosphingolipid lactotetraosylceramide (Lc4cer) did not (Fig. 4A). To investigate the relevance of this effect, we carried out experiments with energy-depleted cells, because under these conditions cellular machineries cannot pinch off vesicles, which previously led to easily visible membrane invaginations when other lipid-binding lectins like Shiga toxin were applied (42, 43). Indeed, LecB was able to induce plasma membrane invaginations in energy-depleted MDCK cells (Fig. 4B). Importantly, β1-integrin colocalized with fluorescently labeled LecB at invaginations (Fig. 4B, magnification), thus implying that LecB can recruit integrins to invaginations. Furthermore, we observed that basolateral LecB application led to marked clustering of endogenous galectin-3 (Fig. S4), which could suggest that LecB outcompetes galectin-3-integrin interaction.

10.1128/mBio.03260-19.4 FIG S4 Control experiments related to Fig. 4, part 2. LecB was applied basolaterally to polarized MDCK cells grown on transwell filters. After fixation, endogenous galectin-3 (green), β1-integrin (red), and nuclei (blue) were stained. Representative confocal sections (*x-y* sections) from a *z*-level 3 μm above the transwell filter surface are displayed. Download FIG S4, PDF file, 1.0 MB. Copyright © 2020 Thuenauer et al. 2020 Thuenauer et al. This content is distributed under the terms of the Creative Commons Attribution 4.0 International license.

In summary, LecB is able to cause membrane invaginations upon binding to fucose-bearing glycosphingolipids. Since LecB is also able to bind integrins and thus to cross-link lipids in membrane invaginations with integrins, this provides a mechanistic explanation for LecB-mediated integrin internalization.

### LecB inhibits cell migration and epithelial wound healing.

**FIG 5:**
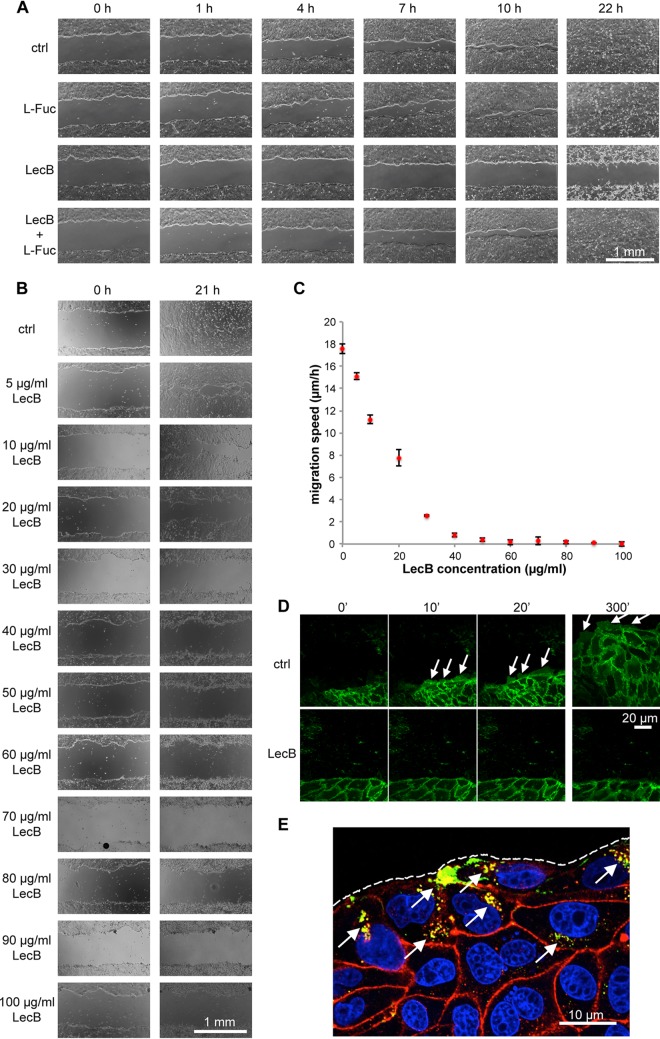
LecB inhibits epithelial wound healing. (A to C) Polarized monolayers of MDCK cells grown in 12-well plates were wounded with a pipette tip and imaged with a wide-field microscope at the indicated time points to observe wound closure. In panel A, cells were treated with LecB and/or l-fucose (43 mM) to block LecB, whereas in panel B increasing concentrations of LecB were used. The quantification of the migration speeds of the wound edges from the latter experiment (C) shows that concentrations larger than 50 μg/ml LecB completely inhibit wound healing. *n* = 3. (D) Polarized monolayers of MDCK cells stably expressing the plasma membrane marker ML-GFP (green) grown on chambered cover glasses were wounded and left untreated (ctrl) or treated with LecB followed by live imaging of the wound edge by confocal microscopy. Lamellipodia are indicated with arrows. (E) Polarized MDCK monolayers grown on chambered cover glasses were wounded and treated with LecB-Alexa Fluor 488 (green) for 3 h. Cells were fixed and stained for β1-integrin (red). An *x-y* confocal section at half height of the cells is shown. Arrows point to internalized β1-integrins colocalizing with LecB-Alexa Fluor 488; the dashed line outlines the wound edge.

As an opportunistic pathogen, P. aeruginosa mainly relies on, and exploits, extrinsic circumstances—like a wound—to gain access to the basolateral side of epithelia. In addition, integrin blocking, e.g., through antibodies, has been previously shown to inhibit cell migration in wound healing assays (44). These considerations motivated us to investigate the effect of LecB on epithelial wound healing. Indeed, the presence of LecB strongly inhibited collective cell migration and wound healing in MDCK monolayers (Fig. 5A). Importantly, this effect was blockable with l-fucose, demonstrating that LecB needs to bind to host cells to cause migration defects. Moreover, we established that the blockage of wound healing by LecB occurred in a dose-dependent manner, with concentrations larger than 50 μg/ml completely blocking cell migration (Fig. 5B and C), whereas another fucose-binding lectin, UEA-I (50 μg/ml), did not induce suppression of wound healing (Fig. S5A). The inhibitory effect of LecB on wound healing was—like other LecB-mediated effects before—reversible by washing out LecB (Fig. S5B).

10.1128/mBio.03260-19.5 FIG S5 Control experiments related to Fig. 5, part 1. (A) Wound healing assays with monolayers of MDCK cells treated with 50 μg/ml UEA-I or LecB. (B) Wound healing assays with monolayers of MDCK cells in which LecB was washed out again as indicated. (C) Regeneration of cell migration at the wound edge of MDCK cells stably expressing ML-GFP (green) treated for 6 h with LecB and then washed out for the indicated times. Download FIG S5, PDF file, 0.7 MB. Copyright © 2020 Thuenauer et al. 2020 Thuenauer et al. This content is distributed under the terms of the Creative Commons Attribution 4.0 International license.

To explain the abrogation of cell migration by LecB, we carried out live-cell imaging experiments with MDCK cells stably expressing the plasma membrane marker ML-GFP (GFP tagged with a Lyn-derived myristoylation motif) (45). ML-GFP allowed visualization of lamellipodia formed by migrating MDCK cells (Fig. 5D, ctrl, arrows). Interestingly, when cells were treated with LecB right after wounding, no lamellipodia formed (Fig. 5D, LecB), whereas washout of LecB was sufficient to reinstate lamellipodium formation and cell migration (Fig. S5C). When migrating cells were treated with LecB, lamellipodia “froze” and LecB strongly bound to lamellipodia (Fig. S6). It is also interesting that cells deeper within the monolayer, which expose only their apical membranes to LecB in this assay, did still move (Fig. S6). In subsequent experiments, we stained for β1-integrins in wound healing assays. This revealed that cells at the wound edge take up large amounts of LecB, and in the same cells, pronounced β1-integrin internalization was evident (Fig. 5E, arrows), which can explain why these cells are not able to migrate any more.

10.1128/mBio.03260-19.6 FIG S6 Control experiments related to Fig. 5, part 2. MDCK cells stably expressing ML-GFP (green) were wounded and observed with a confocal microscope. After 30 min, LecB-Cy3 (red) was added to one sample, whereas the other sample was left untreated (ctrl). Download FIG S6, PDF file, 0.5 MB. Copyright © 2020 Thuenauer et al. 2020 Thuenauer et al. This content is distributed under the terms of the Creative Commons Attribution 4.0 International license.

Taken together, LecB inhibits epithelial wound healing in a reversible manner, which is presumably caused by the fact that integrins in wound edge cells are accessible by LecB and are internalized.

### Depending on LecB expression, P. aeruginosa is able to crawl underneath cells.

**FIG 6:**
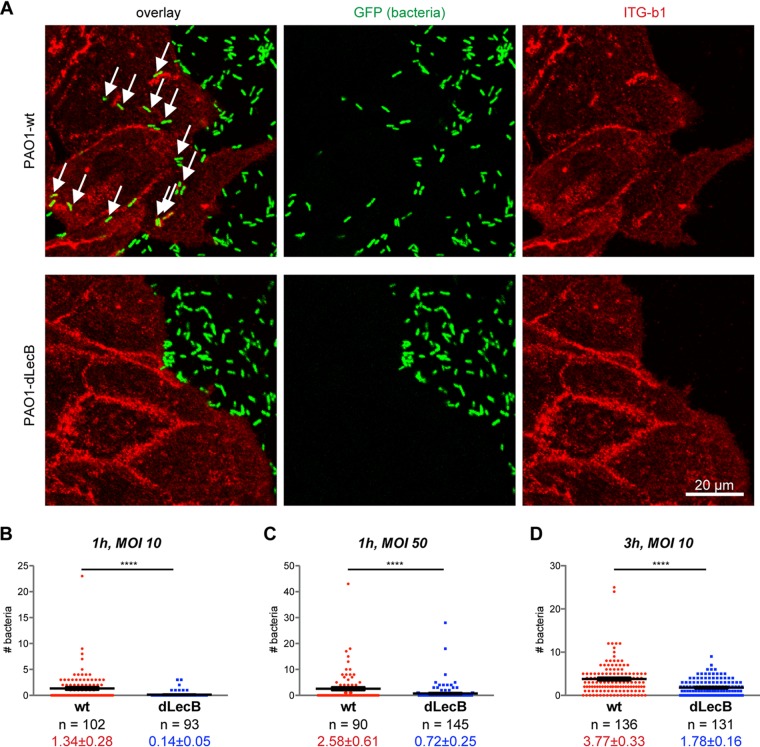
LecB promotes crawling of P. aeruginosa underneath cells. (A) Sparsely seeded MDCK cells were incubated with GFP-tagged PAO1-wt or LecB-deficient PAO1-dLecB (green) at an MOI of 50 for 1 h. After fixation, β1-integrins were stained in red. For each condition, a confocal *x-y* section at the level of cell adhesion to the substrate, which was taken from a complete three-dimensional confocal stack, is displayed. Arrows indicate bacteria underneath cells. (B to D) After carrying out an experiment as described for panel A but with the indicated MOIs and incubation periods, the numbers of bacteria underneath cells were determined per cell. Each data point represents an individual cell, and the black lines indicate the mean and the SEM. For evaluating statistical significance, Mann-Whitney testing using GraphPad Prism 5 was applied; ****, *P* < 0.0001.

The additional and probably dominant cytotoxic effects caused by the numerous toxins produced by P. aeruginosa prevented us from directly quantifying an effect of LecB knockdown in *in vitro* wound healing assays with live P. aeruginosa. However, we observed that P. aeruginosa (PAO1-wt) is able to crawl underneath exposed cells (Fig. 6A). We postulated that this requires at least local loosening of potentially integrin-mediated cell-substrate adhesion. We tested this hypothesis by investigating the influence of LecB on “P. aeruginosa crawling.” To this end, we used a LecB-deficient PAO1 strain (PAO1-dLecB), which exhibited the same growth kinetics as PAO1-wt (Fig. S7A). After overnight culture, the PAO1-wt strain showed clear expression of LecB, whereas the LecB-deficient P. aeruginosa strain (PAO1-dLecB) did not (Fig. S7B and C). In accordance with our hypothesis, PAO1-dLecB was visibly found in smaller numbers underneath cells (Fig. 6A). To substantiate the experimental procedure, we established that increasing the multiplicity of infection (MOI) (Fig. 6B and C) and increasing the duration of incubation (Fig. 6B and D) also increased the number of bacteria crawling underneath cells. Importantly, for all investigated conditions, the number of bacteria crawling underneath per cell was lower for the PAO1-dLecB strain than the PAO1-wt strain.

10.1128/mBio.03260-19.7 FIG S7 Control experiments related to Fig. 6. (A) OD_600_ growth curves from wt PAO1 (PAO1-wt) and LecB-knockout (PAO1-dLecB) strains grown in LB medium at 37°C. (B and C) The presence of LecB was probed in PAO1-wt and PAO1-dLecB, both stably expressing GFP, by WB (B) and semiquantitative real-time PCR with *rpoD* as a reference (C). Download FIG S7, PDF file, 0.2 MB. Copyright © 2020 Thuenauer et al. 2020 Thuenauer et al. This content is distributed under the terms of the Creative Commons Attribution 4.0 International license.

In summary, our study reveals a novel mechanism in which LecB, through impairing cell-to-basement membrane attachment, allows for the creation of microniches enabling subepithelial colonization by P. aeruginosa.

## DISCUSSION

### Integrins are major receptors of LecB.

Lectins bind to carbohydrates and can therefore have multiple receptors that express the appropriate glycosylation. Hence, it is interesting that our experiments suggest a favored binding of LecB to integrins. LecB binding to β1-integrin appeared to be strong, because LecB was able to recover 75% of total β1-integrin from cells when it was basolaterally applied and had access to β1-integrin only at the cell surface. As expected, LecB binding to β1-integrin solely occurred through carbohydrates, since removal of β1-integrin glycosylation abolished binding. Furthermore, mass spectrometry analysis of all basolateral LecB receptors revealed that virtually all integrins expressed by MDCK cells were among the top hits, including many integrin-associated proteins that were presumably recovered through coprecipitation with integrins.

This introduces LecB as a new member of the list of bacterial molecules that bind integrins. Another fucose-binding lectin, UEA-I, was not able to cause any of the cellular effects that LecB caused. This shows that binding to fucose is not sufficient by itself and suggests that LecB has additional features that bring about its specific capabilities. First, LecB and UEA-I prefer different fucose linkages in oligosaccharides (UEA-I binds α_1,2_-linked fucose but only weakly binds α_1,3_- and α_1,6_-linked fucose [46, 47]; LecB shows a slight preference for α_1,4_-linked fucose but also binds to α_1,2_- and α_1,3_-linked fucose [48–50]). In addition, LecB is a tetramer with four fucose-binding sites, but UEA-1 is a dimer offering only two binding sites (35).

It is also worth noting that the sequence of LecB varies slightly between different strains of P. aeruginosa (51, 52). Since ligand binding among LecB variants is conserved, we expect that these LecB variants are utilized in similar ways as we reported here for LecB in the P. aeruginosa strain PAO1.

### Mechanism of LecB-mediated integrin internalization.

Since LecB recognizes only the carbohydrate moieties of integrins, it is able to manipulate integrins via unique mechanisms. The structure of LecB with four opposing binding sites is ideal to cross-link receptors. This has been demonstrated before *in vitro* by showing that LecB is able to cross-link GUVs that contain LecB receptors (53).

We hypothesize that the cross-linking capacity of LecB is key for the observed rapid internalization of integrins. In addition to integrins, LecB was also able to recognize fucose-bearing glycosphingolipids and to cause membrane invaginations without the need of additional energy input, which replicates effects caused by other glycosphingolipid-binding lectins like Shiga toxin subunit B (StxB) (42), cholera toxin subunit B 9 (CtxB) (54), or *Ralstonia solanacearum* lectin (RSL) (55). In addition, β1-integrin was also found on LecB-generated membrane invaginations on energy-depleted cells. This suggests that LecB on the one hand causes membrane invaginations and on the other hand is able to recruit integrins to these invaginations, thus constituting a potent endocytic mechanism. A similar mechanism was suggested for the host cell-endogenous protein galectin-3 (40). This means that bacteria have evolved molecules like LecB that can hijack this endogenous uptake route. Importantly, LecB-mediated lipid-integrin cross-linking for internalization represents a mechanism that can explain the observed integrin internalization that occurred regardless of integrin activation status and carried also basement membrane ligands like laminins with it.

### LecB-triggered inhibition of wound healing.

LecB binding to basolateral cell surfaces caused cellular effects that could be causally linked to integrin internalization. In fully polarized epithelial cells, binding of LecB to basolateral cell surfaces but not to apical cell surfaces, which contain only minute amounts of integrins, led to loss of apicobasal polarity. This indicates the need of integrin internalization for dissolution of polarity. In addition, loss of polarity was reversible after washout of LecB and coincided well with the return of β1-integrin to the basolateral plasma membrane. Our data also suggest that LecB-mediated integrin internalization is responsible for the observed block in cell migration in epithelial wound healing assays. First, integrins were preferentially internalized in wound edge cells, and LecB prominently bound to lamellipodia in edge cells. This makes sense, since edge cells offer more cellular surface area for LecB binding and expose their integrins, in contrast to cells deeper within the intact monolayer, which display only their apical surfaces to LecB. Second, edge cells rapidly and efficiently stopped moving upon LecB treatment, whereas other cells deeper within the monolayer preserved their capacity to move within the monolayer.

The effects that LecB exerts through integrin internalization require basolateral access of LecB. In this context, it is interesting that P. aeruginosa possesses strategies to convert apical into basolateral plasma membranes (34). However, the direct way for P. aeruginosa and LecB to access integrins is through wounds in an epithelial cell layer.

P. aeruginosa preferentially colonizes wounds, and diminished healing of P. aeruginosa-infected wounds was reported previously (10). Importantly, P. aeruginosa possesses multiple mechanisms to manipulate and to intoxicate host cells. We therefore anticipate that, in order to inhibit wound healing, P. aeruginosa will apply different combinations of its arsenal, including LecB-mediated epithelial depolarization and inhibition of cell migration, depending on the host tissue (25).

### LecB has a role in enabling bacteria to crawl underneath host cells.

Our experiments revealed a novel feature of P. aeruginosa behavior. We observed that bacteria frequently crawled underneath host cells. For this, at least local loosening of cell-substrate adhesion is required, which could be achieved by LecB-mediated integrin internalization. We therefore investigated the contribution of LecB to crawling of the P. aeruginosa strain PAO1 underneath cells. Indeed, knocking out LecB significantly decreased crawling events. Based on our results, we suggest that P. aeruginosa uses LecB to manipulate integrin-basement membrane interaction to proceed along the interface between epithelial cells and the basement membrane.

In summary, our work brings integrins into focus as targets of P. aeruginosa and provides additional rationales for the ongoing efforts to develop LecB inhibitors as a treatment strategy in addition to antibiotics (26–31).

## MATERIALS AND METHODS

### Antibodies, plasmids, and reagents.

Used antibodies are listed in Table S2 in the supplemental material. The plasmid pPH-Akt-GFP encoding PH-Akt-GFP was a gift from Tamas Balla (Addgene plasmid no. 51465). The plasmid encoding GFP tagged with a glycosylphosphatidylinositol (GPI) anchor (pGPI-GFP) was described before (56). The plasmid encoding GFP tagged with a Lyn-derived myristoylation motif (pML-GFP) was a gift from Christian Wunder (Curie Institute, Paris, France).

10.1128/mBio.03260-19.9 TABLE S2 Lists of primary and secondary antibodies used. WB, Western blot; IF, immunofluorescence; IP, immunoprecipitation; surf. stain, used for surface staining in live cells; milk, milk used as blocking agent; methanol, cell fixation with methanol. Download Table S2, PDF file, 0.2 MB. Copyright © 2020 Thuenauer et al. 2020 Thuenauer et al. This content is distributed under the terms of the Creative Commons Attribution 4.0 International license.

Recombinant LecB was produced in Escherichia coli BL21(DE3) cells and purified with affinity columns as previously described (41). LecB and fluorophore-conjugated LecB were used at a concentration of 50 μg/ml (4.3 μM) unless stated otherwise. UEA-I and UEA-I–fluorescein isothiocyanate (FITC) were from Vector Labs. Cycloheximide, and l-fucose (6-deoxy-l-galactose) were from Sigma-Aldrich.

### Mammalian cell culture and creation of stable cell lines.

MDCK strain II cells were cultured in Dulbecco’s modified Eagle’s medium (DMEM) supplemented with 5% fetal calf serum (FCS) at 37°C and 5% CO_2_. Unless stated otherwise, 3 × 10^5^ MDCK cells were seeded per transwell filter (0.4-μm pore size, polycarbonate membrane, catalog no. 3401 from Corning) and cultured for 4 days before experiments. For the creation of stable MDCK cell lines, plasmids encoding the proteins of interest and G418 resistance (pPH-Akt-GFP, pML-GFP, and pGPI-GFP) were transfected into cells with Lipofectamine 2000 (Thermo Fisher). After the cells were allowed to express the proteins overnight, they were trypsinized and plated sparsely in medium containing 1 mg/ml G418. After single colonies had formed, GFP-positive colonies were extracted with cloning rings. At least 6 colonies were extracted for each cell line, grown on transwell filters for 4 days, fixed, and stained against the basolateral marker protein β-catenin and the tight junction marker protein ZO-1 to assay their polarized morphology. Based on these results, we chose one colony for each cell line for further experiments. TEER measurements were carried out using an EVOM2 equipped with chopstick electrodes (World Precision Instruments).

### Immunofluorescence.

Cells were washed two times with phosphate-buffered saline without Ca^2+^ and Mg^2+^ (PBS) and then fixed with 4% (wt/vol) formaldehyde (FA) for 15 min at room temperature. Samples were treated with 50 mM ammonium chloride for 5 min to quench FA and then permeabilized with a SAPO medium (PBS supplemented with 0.2% [wt/vol] bovine serum albumin [BSA] and 0.02% [wt/vol] saponin) for 30 min. Primary antibodies were diluted in SAPO medium and applied on the samples for 60 min at room temperature. After three washes with PBS, secondary dye-labeled antibodies and, if required, 4′,6-diamidino-2-phenylindole (DAPI) and dye-labeled phalloidin were diluted in SAPO medium and applied to the cells for 30 min at room temperature (details for the antibodies used are listed in Table S2). After 5 washes with PBS, cells were mounted for microscopy. Since α3-integrin antibodies did not work in FA-fixed cells, methanol fixation was applied in this case. Briefly, cells were incubated with precooled methanol for 15 min at −20°C. After washing with PBS, cells were permeabilized with 0.05% (vol/vol) Triton X-100 for 10 min at room temperature and blocked with 3% (wt/vol) bovine serum albumin (BSA) for 1 h at room temperature. Staining with primary and secondary antibodies was then carried out as described before but with a 3% (wt/vol) BSA solution.

### Microscopic imaging of fixed cells and live-cell experiments.

For microscopic imaging, an A1R confocal microscope (Nikon) equipped with a 60× oil immersion objective (numerical aperture [NA] = 1.49) and laser lines at 405 nm, 488 nm, 561 nm, and 641 nm was utilized. Image acquisition and analysis were performed with NIS-Elements 4.10.04 (Nikon).

Live-cell experiments were carried out at 37°C, and MDCK cells grown as polarized monolayers for 3 days on Lab-Tek II chambered cover glasses (8 well, 1.5 borosilicate glass) were used. The medium was changed to recording medium (Hanks’ balanced salt solution [HBSS] supplemented with 1% FCS, 4.5 g/liter glucose, and 20 mM HEPES).

### Wound healing assays.

MDCK cells were seeded on 12-well plates or, for live-cell microscopy of cell migration, on 8-well Lab-Tek II chambered cover glasses and allowed to form confluent monolayers for 3 days. Then, cells were scratched with a 200-μl pipette tip to inflict a wound. On 12-well plates, marker lines were drawn on the bottom to ensure that always the same position of the wound was imaged.

### Western blot analysis.

Cells were washed twice with PBS and lysed in RIPA buffer (20 mM Tris [pH 8], 0.1% [wt/vol] SDS, 10% [vol/vol] glycerol, 13.7 mM NaCl, 2 mM EDTA, and 0.5% [wt/vol] sodium deoxycholate in water), supplemented with protease inhibitors (0.8 μM aprotinin, 11 μM leupeptin, 200 μM Pefabloc) and phosphatase inhibitor (1 mM sodium orthovanadate). Protein concentrations were analyzed using a bicinchoninic acid (BCA) assay kit (Pierce). Equal amounts of protein per lysate were separated by SDS-PAGE and transferred to a nitrocellulose membrane. The membrane was blocked with Tris-buffered saline (TBS) supplemented with 0.1% (vol/vol) Tween 20 and 3% (wt/vol) BSA for 1 h and incubated with primary and horseradish peroxidase (HRP)-linked secondary antibodies diluted in the blocking solution. In some cases, TBS supplemented with 0.1% (vol/vol) Tween 20 and 5% (wt/vol) milk powder was used (details for the antibodies and conditions used are listed in Table S2). Detection was performed by a chemiluminescence reaction using the Fusion-FX7 Advance imaging system (Peqlab Biotechnologie GmbH).

### Energy depletion.

Energy depletion was carried out as described before (42). Briefly, MDCK cells were washed 2 times with PBS supplemented with 100 mg/liter CaCl_2_ and 100 mg/liter MgCl_2_·6H_2_O (PBS^++^) and then treated with PBS^++^ supplemented with 10 mM 2-deoxy-d-glucose and 10 mM NaN_3_ for 30 min at 37°C.

### Bacterial culture and crawling experiments.

GFP-tagged P. aeruginosa PAO1 wild-type (PAO1-wt) and LecB-deficient (PAO1-dLecB) strains were provided by S. de Bentzmann (CNRS, Aix Marseille University, France). The generation of LecB-deficient PAO1 is described in reference 23, and GFP tagging was carried out according to the procedure described in reference 57. For experiments, bacteria were cultured overnight (approximately 16 h) in LB-Miller medium containing 60 μg/ml gentamicin in a shaker (Thriller; Peqlab) at 37°C and 650 rpm. The bacteria reached an optical density (OD) measured at 600 nm of approximately 5. Using these growth conditions, PAO1-wt and PAO1-dLecB strains showed comparable growth kinetics (Fig. S7A) and harvested PAO1-wt efficiently expressed LecB, whereas PAO1-dLecB did not (Fig. S7B and C).

For crawling experiments, MDCK cells were sparsely seeded on 8-well Lab-Tek II chambered cover glasses and cultured for 1 day, so that clusters of maximally 10 cells in diameter formed to ensure that all cells were exposed to P. aeruginosa similarly as at a wound edge. Then, PAO1-wt or PAO-dLecB was applied for the indicated MOI and duration. Bacteria crawling under cells were counted manually per cell from confocal image stacks of whole cells to ensure that only bacteria located directly underneath cells at the level of the glass coverslip were counted.

### qPCR.

PAO1-wt and PAO1-dLecB were cultured overnight as described before. RNA was extracted using TRI reagent (Sigma-Aldrich). After DNase digest, 100 ng RNA was transcribed into cDNA using a first-strand cDNA synthesis kit (Thermo Fisher). Then, quantitative PCR (qPCR) was performed on a CFX384 qPCR cycler (Bio-Rad) using a SYBR Select master mix (Thermo Fisher) and the following primers: for LecB, forward 3′-AAGGAGTGTTCACCCTTCCC-5′ and reverse 3′-GATGACGGCGTTATTGGTGC-5′; for *rpoD* as reference, forward 3′-GGGATACCTGACTTACGCGG-5′ and reverse 3′-GGGGCTGTCTCGAATACGTT-5′.

### Labeling of lectins.

LecB was labeled with fluorescent dyes bearing *N*-hydroxysuccinimide (NHS) esters as reactive groups (Cy3 monoreactive NHS ester [GE Healthcare], Cy5 monoreactive NHS ester [GE Healthcare], Alexa Fluor 488 NHS ester [Thermo Fisher]) or with biotin using NHS-polyethylene glycol 12-biotin (Thermo Fisher) according to the instructions of the manufacturers and purified using PD-10 desalting columns (GE Healthcare).

### Cell surface biotinylation and immunoprecipitation.

For cell surface biotinylation, all following steps were carried out in a cold room (4°C). Sulfo-NHS-biotin (Thermo Fisher) was freshly diluted in PBS^++^ (concentration, 0.3 mg/ml) and applied to the apical or basolateral plasma membrane of transwell filter-grown MDCK cells for 20 min. Afterward, the reaction was quenched for 20 min with PBS^++^ supplemented with 50 mM ammonium chloride. Cells were lysed with RIPA buffer, and biotinylated proteins were precipitated with streptavidin-agarose beads (Thermo Fisher). Elution was carried out with Laemmli buffer (2% [wt/vol] SDS, 10% [vol/vol] glycerol, 60 mM Tris-Cl [pH 6.8] in water) and boiling at 98°C for 5 min.

For β1-integrin immunoprecipitation (IP), MDCK cells were grown to confluence in 10-cm dishes and lysed in IP lysis buffer (50 mM Tris-HCl [pH 7.5], 150 mM sodium chloride, 1% [vol/vol] IGEPAL CA-630, 0.5% [wt/vol] sodium deoxycholate in water). The lysates were precleared with protein A-agarose beads (Roche) for 3 h and then incubated with anti-β1-integrin antibodies (MAB2000 from Millipore) for 1 h. After addition of protein A-agarose beads overnight, beads were washed three times with IP-lysis buffer, and β1-integrin was eluted with Laemmli buffer and boiling at 98°C for 5 min.

### Mass spectrometry-based identification of LecB interaction partners.

MDCK cells were cultured in medium for stable-isotope labeling by amino acids in cell culture (SILAC medium) for 9 passages and then seeded on transwell filters and allowed to polarize for 4 days. For the first sample, biotinylated LecB was applied to the apical side of light-SILAC-labeled cells and on the basolateral side of medium-SILAC-labeled cells, whereas heavy-SILAC-labeled cells received no stimulation and served as control. For the second sample, the treatment conditions were permuted. After lysis with IP lysis buffer, the different SILAC lysates were combined and LecB-biotin-receptor complexes were precipitated using streptavidin-agarose beads as described before. Eluted LecB-biotin-receptor complexes were then prepared for mass spectrometry (MS) analysis using SDS-PAGE gel electrophoresis. Gels were cut into pieces, proteins therein were digested with trypsin, and resulting peptides were purified by stop-and-go-extraction (STAGE) tips.

For mass spectrometry analysis, samples were fractionated by nanoscale high-pressure liquid chromatography (HPLC) on a 1200 HPLC (Agilent Technologies, Waldbronn, Germany) connected online to an LTQ Orbitrap XL mass spectrometer (Thermo Fisher Scientific, Bremen, Germany). Fused silica HPLC-column tips with a 75-μm inner diameter were self-packed with ReproSil-Pur 120 ODS-3 (Dr. Maisch, Ammerbuch, Germany) to a length of 20 cm. Samples were directly injected into the mass spectrometer (for details see reference 58). The raw data files were uploaded into the MaxQuant software. Database searches were performed against a full-length dog database containing common contaminants such as keratins and enzymes used for in-gel digestion. Carbamidomethylcysteine was set as fixed modification, and oxidation of methionine and protein amino-terminal acetylation were set as variable modifications. Triple SILAC was used as quantitation mode. The enzyme specificity was trypsin/P+DP with three allowed miscleavages. The MS/MS tolerance was set to 0.5 Da, and the mass precision of identified peptides after recalibration was in general less than 1 ppm. For identification and quantitation, the following settings were used: peptide and protein false-discovery rates (FDR) were set to 0.01; maximum peptide posterior error probability (PEP) was set to 0.1; minimum peptide length was set to 7; minimum number peptides for identification and quantitation of proteins was set to two, of which one must be unique; minimum ratio count was set to two; and only unmodified peptides and the variable modification were used for protein quantification. The “match between run” option was used with a time window of 2 min.

From the generated list of MS-identified proteins, we defined those proteins as LecB interaction partners that showed more than 2-fold enrichment on a log_2_ scale over controls in both SILAC samples (see Table S1 in the supplemental material).

### GUV experiments.

Giant unilamellar vesicles (GUVs) were composed of 1,2-dioleoyl-*sn*-glycero-3-phosphocholine (DOPC), spiked with 1 mol% 2-(4,4-difluoro-5,7-dimethyl-4-bora-3a,4a-diaza-*s*-indacene-3-pentanoyl)-1-hexadecanoyl-*sn*-glycero-3-phosphocholine (β-BODIPY FL C_5_-HPC), 30 mol% cholesterol, and 5 mol% of the desired glycosphingolipid species. Blood group glycosphingolipids were provided by Göran Larson (Sahlgrenska University Hospital, Gothenburg, Sweden). GUVs were grown at room temperature using the electroformation technique on indium-tin oxide (ITO)-coated slides as described previously (42, 43). Briefly, lipid mixtures were dissolved in chloroform at a final concentration of 0.5 mg/ml, and 15 μl solution was spread on the conductive surface of ITO slides. After 2 h of drying under vacuum, GUVs were grown in a 290 mosM sucrose solution by applying an alternating electric field from 20 mV to 1.1 V for 3 h.

LecB-Cy3 (15 μg/ml) was incubated with GUVs at room temperature and examined under an inverted confocal fluorescence microscope (Nikon A1R) equipped with an oil immersion objective (60×; NA, 1.49).

### Statistics.

If not stated otherwise, data obtained from *n* = 3 independent experiments were used to calculate arithmetic means. Error bars represent standard errors of the means (SEM). Statistical significance analysis was carried out using GraphPad Prism 5.
