# Supplementary material for: The Pseudomonas aeruginosa Lectin LecB Causes Integrin Internalization and Inhibits Epithelial Wound Healing
Source: mBio. 2020 Mar 10;11(2):e03260-19. doi: 10.1128/mBio.03260-19 (PMC7064779; doi:10.1128/mBio.03260-19)
Supplement: FIG S1 [file mBio.03260-19-sf001.pdf]

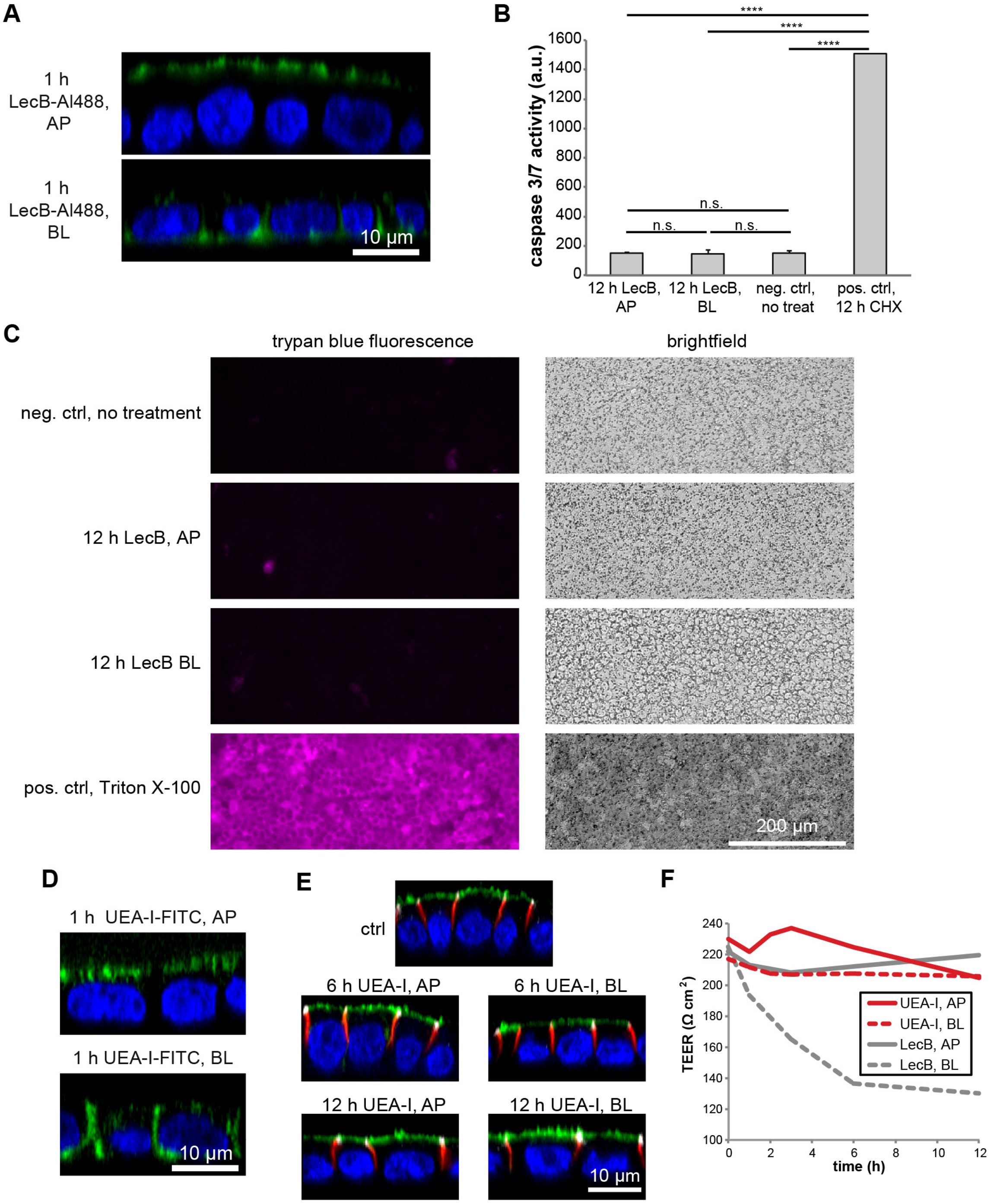

Figure S1: Control experiments related to Fig. 1

(A) 50 µg/ml LecB-Alexa488 (green) was applied to polarized filter-grown MDCK cells apically (AP) or basolaterally (BL). After fixation, nuclei were stained with DAPI (blue). Confocal x-z sections are displayed. (B) MDCK cells were left untreated as negative control, treated with 50 µg/ml LecB as indicated, or treated with cycloheximide (CHX; 100 µg/ml) as positive control. After cell lysis a Caspase-Glo 3/7 assay (Promega) was performed to measure the level of induced apoptosis. For evaluating statistical significance, one-way ANOVA with Tukey's post hoc test using GraphPad Prism 5 was applied, **** denotes $p < 0.0001$, n.s. denotes $p > 0.05$. (C) Polarized filter-grown MDCK cells were left untreated (neg. ctrl), treated AP or BL with LecB for 12 h, or treated with 0.1% Triton X-100 for 5 min (pos. ctrl.). Afterwards cells were incubated AP and BL for 5 min with 0.4% trypan blue and then washed 2 times with PBS. Since trypan blue is fluorescent upon excitation with far-red

light, its presence in cells was measured using a wide field microscope equipped with a far-red (Cy5)-cube (magenta). Cells were also imaged in brightfield mode. In these images the pores of the transwell filters are visible as small black dots. (D) 50 μg/ml UEA-I-FITC (green) was applied to polarized filter-grown MDCK cells apically (AP) or basolaterally (BL). After fixation nuclei were stained with DAPI (blue). Confocal x-z sections are displayed. (E) Polarized, filter-grown MDCK cells stably expressing the apical marker GPI-GFP (green) were left untreated (ctrl) or treated apically (AP) or basolaterally (BL) with 50 μg/ml UEA-I for the indicated time periods, fixed and stained with antibodies recognizing the basolateral marker β-catenin (red) and the tight junction marker ZO-1 (white), nuclei were stained with DAPI (blue). Representative confocal x-z sections are shown. (F) MDCK cells were treated with 50 μg/ml UEA-I as indicated and the trans-epithelial electrical resistance (TEER) was measured. As comparison the data from treating cells with LecB from Fig. 1B are shown in gray.
