# Supplementary material for: The Pseudomonas aeruginosa Lectin LecB Causes Integrin Internalization and Inhibits Epithelial Wound Healing
Source: mBio. 2020 Mar 10;11(2):e03260-19. doi: 10.1128/mBio.03260-19 (PMC7064779; doi:10.1128/mBio.03260-19)
Supplement: FIG S2 [file mBio.03260-19-sf002.pdf]

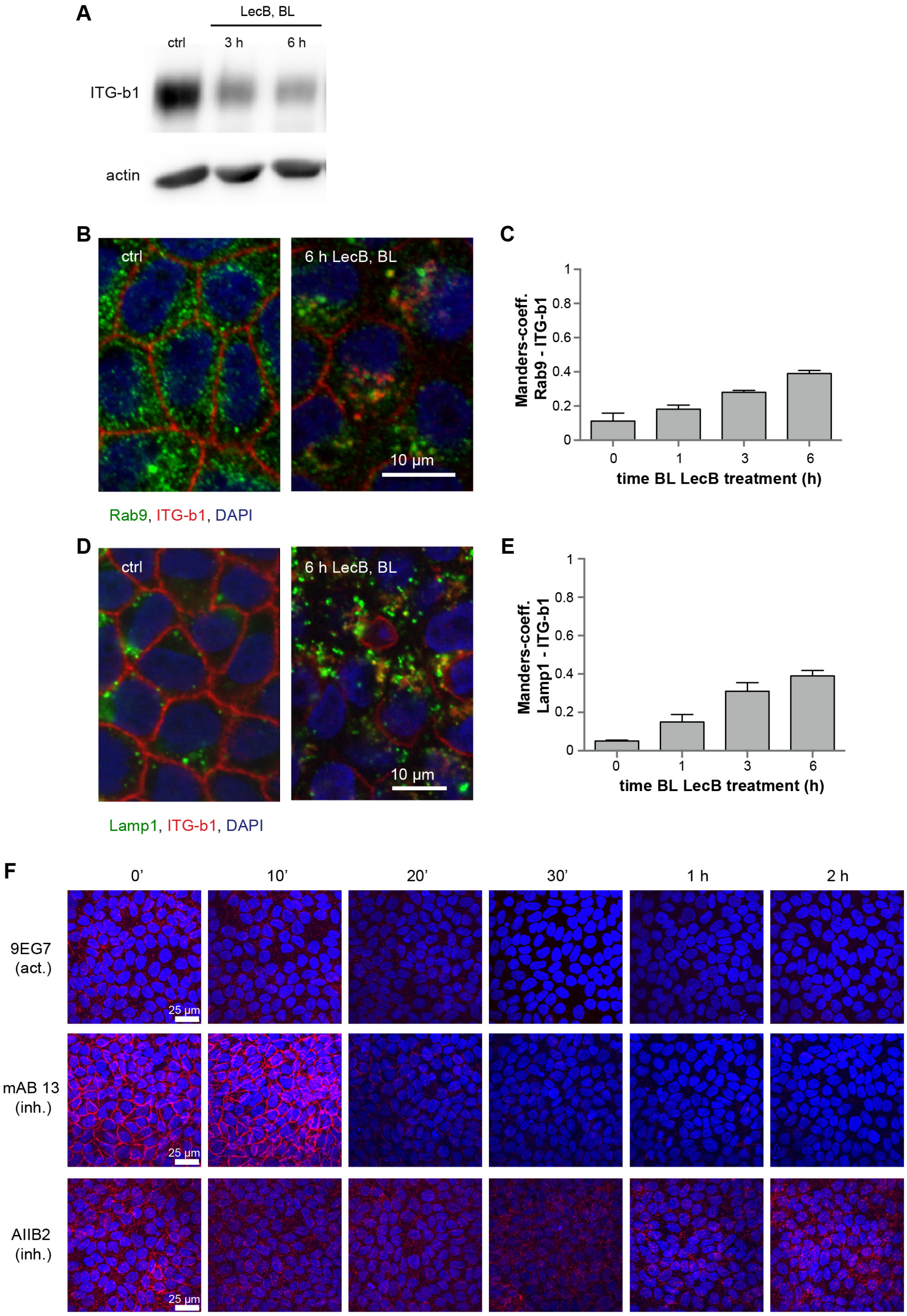

Figure S2: Control experiments related to Fig. 3

(A) The total amounts of β1-integrin were probed by WB in MDCK cells basolaterally treated with LecB. (B) – (C) MDCK cells were basolaterally treated with LecB as indicated, fixed, stained for Rab9 (green), β1-integrin (red), and nuclei were stained with DAPI (blue). Representative confocal sections (x-y sections) through the middle of the cells are depicted in (B) and the quantification of the Manders overlap-coefficient between Rab9 and β1-integrin from n = 3 independent experiments is depicted in (C). (D) – (E) MDCK cells were basolaterally treated with LecB as indicated, fixed, stained for Lamp1 (green), β1-integrin (red), and nuclei were stained with DAPI (blue). Representative confocal sections (x-y sections) through the middle of the cells are shown in (D) and

the quantification of the Manders overlap-coefficient between Lamp1 and β1-integrin from n = 3 independent experiments is depicted in (E). (F) LecB was applied basolaterally to polarized filter-grown MDCK cells for the indicated time periods followed by basolateral application of activation-specific anti-β1-integrin antibodies (9EG7, mAB 13, AIIB2) to live cells. After fixation, the signal from bound anti-β1-integrin antibodies was measured with a confocal microscope. Representative maximum intensity projections of confocal image stacks covering full cell heights are displayed.
