# Supplementary material for: The Pseudomonas aeruginosa Lectin LecB Causes Integrin Internalization and Inhibits Epithelial Wound Healing
Source: mBio. 2020 Mar 10;11(2):e03260-19. doi: 10.1128/mBio.03260-19 (PMC7064779; doi:10.1128/mBio.03260-19)
Supplement: TABLE S1 [file mBio.03260-19-st001.pdf]

Table S1: List of basolateral LecB interaction partners identified by SILAC MS

#	Protein IDs	Gene name	Number of proteins	Peptides	PEP	log2 (baso vs ctrl) 01	log2 (baso vs ctrl) 02
1	E2QSC8	TSPAN8	1	2	1,67E-29	6,61	5,86
2	F1PP33;J9NUT8	ITGA1	2	12	1,38E-73	5,97	3,14
3	F1PR26;J9NW82	PTGFRN	2	36	7,14E-133	5,82	4,86
4	E2RBL9	ITGB6	1	12	2,41E-46	5,74	4,82
5	E2QZT4	BSG	1	11	2,74E-37	5,52	5,14
6	E2RT60	ITGB1	1	18	1,23E-132	5,47	6,33
7	J9PB47;A1YV64	CAECAM1; CEACAM28	2	4	9,72E-38	5,36	4,52
8	O97702;F1PPG5	GPIIIa;ITGB3	2	13	6,15E-74	5,24	5,18
9	F1PF03;E2RH87	EGFR	2	16	4,79E-76	5,22	2,85
10	E2QX93	PCDH1	1	13	8,09E-60	5,21	3,72
11	J9P3V6;F1PWJ7	BCAM	2	20	4,82E-239	5,21	5,26
12	F1PRC5	SLC3A2	1	16	2,43E-64	5,09	3,91
13	B6V8E6	CTNNB1	1	15	1,43E-82	4,98	2,99
14	F1P9W5;E2RRI1	SLC12A2	2	25	1,39E-121	4,95	4,64
15	F1P8Q0	ITGAV	1	33	1,48E-213	4,88	3,72
16	F1PUP2;F1PUN5; F1PTF5;F1PTF3; F1PTF2	PTPRM	5	12	1,19E-41	4,80	2,26
17	P33724;P33724-2; F1PWG1	CAV1	3	3	1,89E-15	4,74	4,61
18	F1PGD5	LY75	1	14	2,78E-52	4,73	3,28
19	J9NZA9;F1PBA1; P50997;F1P767; F1PL53;F1PJF0; P50996;F1PRH6; J9NZ73;J9P483; F1P8N4	ATP1A1	11	36	0,00E+00	4,71	5,06
20	E2R9S7;J9NXR7; F1Q015;F1PG43	CTNNA1	4	21	3,67E-233	4,70	3,48
21	E2RL88;J9NVU0	ITGA6	2	26	2,35E-149	4,69	2,99
22	E2RMT2;F1PHH3	PTK7	2	19	1,86E-112	4,68	3,10
23	E2RFE1	ITGB4	1	17	8,20E-139	4,65	3,48
24	J9NXR3	Uncharacterized protein	1	13	5,23E-63	4,58	3,46
25	F1PTY0;J9PAF7	SLC4A7	2	5	1,53E-15	4,58	2,50
26	F1PAA3	CDH3	1	9	3,27E-23	4,43	4,60
27	P31637	SLC5A3	1	3	1,26E-11	4,41	2,60
28	F1PAA4;F1PAA9	CDH1	2	14	9,61E-100	4,39	4,55
29	E2R2V6	L1CAM	1	36	1,56E-222	4,38	3,80
30	E2R0Z0;E2R858; J9NXY3;E2R971	CDH6	4	16	2,27E-49	4,37	6,21
31	F1PV63	CDCP1	1	11	2,12E-51	4,30	3,38
32	F1P9S5;J9NRH0; J9PA53;E2QY27; E2RDE1	PLXNB2	5	27	4,97E-140	4,24	3,04
33	F1PXU6;J9NY09; E2RPM8;E2R795	IGF1R	4	26	5,53E-110	4,20	4,24

#	Protein IDs	Gene name	Number of proteins	Peptides	PEP	log2 (baso vs ctrl) 01	log2 (baso vs ctrl) 02
34	E2REA9	ITGA2	1	28	1,35E-257	4,14	4,19
35	J9NTL4;F1PK94	CDH17	2	16	9,47E-99	4,07	3,42
36	J9P423;F1PTZ7;Q28284	CD44	3	6	1,56E-18	3,99	6,38
37	E2R4F0;J9P8K7	CELSR2	2	19	1,29E-63	3,83	2,50
38	Q9XSU4;F2Z4N5;J9P7F5	RPS11	3	5	1,14E-09	3,81	3,58
39	F1PU73	NCSTN	1	6	1,11E-23	3,81	4,38
40	Z4YHE9;Q75ZY9;J9P743;F1PEM3;J9NY14;F1PR52;E2RSH8;F6XRA9	MET	8	20	6,25E-84	3,80	3,73
41	F1Q439	ITGA3	1	13	3,28E-122	3,78	5,75
42	E2RE80;E2RE81;F1PH17;E2R6Z2	PTPRF	4	34	4,63E-134	3,75	2,89
43	F1PWL4	PVRL2	1	5	9,06E-24	3,71	2,54
44	F1PGJ7;J9P5N9;E2RNZ4	DDR1	3	12	2,07E-67	3,70	4,70
45	F1PBI6	THBS1	1	11	5,93E-43	3,67	2,54
46	F1PRQ8;F1PAD6	EPHB4	2	10	7,64E-53	3,62	2,39
47	E2QXI6	TPBG	1	5	1,45E-19	3,59	3,32
48	F1PIQ9;O18735;F1PQ05;F1PAC5;J9P9R2;J9P025;E2R7P5;F1P627;E2RHY5;F1P626;J9P9H9	ERBB2	11	17	9,55E-72	3,59	4,80
49	F1Q1M9;F1PM51	EPCAM	2	12	2,05E-75	3,49	3,53
50	F1PFS1	STOM	1	5	6,91E-32	3,28	3,62
51	J9P897;F2Z4P3;Q9XSU3	RPL23	3	4	2,21E-12	3,27	3,29
52	P06583;J9P7J0;F1PMF0	ATP1B1	3	8	3,27E-23	3,25	4,54
53	F1PAF1;E2R7J5	EPHB2	2	10	1,43E-62	3,19	3,11
54	J9P798;J9P7C2;J9NSL1;E2RPP2;J9JHX3;E2RCJ1;E2R443	RPS15A	7	5	6,47E-18	3,12	2,22
55	F1PPI5	SLC1A5	1	5	7,18E-40	2,88	2,82
56	E2RH47	RPS3	1	8	1,60E-55	2,85	2,53
57	E2R9G7;E2QTG3	RPL11	2	3	1,28E-50	2,70	2,49
58	F1P9K1	IGSF3	1	15	8,96E-73	2,63	2,35
59	F1PVS7	PVR	1	8	2,09E-27	2,50	3,56
60	F1Q0H9	SLC1A4	1	4	1,18E-14	2,48	2,83
61	P33729;F1PB95	ICAM1	2	7	6,33E-27	2,44	2,25
62	F1PRL5;E2RRG4	SLC25A5	2	6	1,66E-22	2,27	2,20
63	J9P5A2;F1PLY1	CELSR1	2	10	2,83E-47	2,27	2,85
64	E2R8R8	RPS9	1	4	4,70E-11	2,18	2,39
65	E2R149	RPL9	1	5	9,36E-17	2,04	2,91
