# Supplementary material for: The Pseudomonas aeruginosa Lectin LecB Causes Integrin Internalization and Inhibits Epithelial Wound Healing
Source: mBio. 2020 Mar 10;11(2):e03260-19. doi: 10.1128/mBio.03260-19 (PMC7064779; doi:10.1128/mBio.03260-19)
Supplement: FIG S3 [file mBio.03260-19-sf003.pdf]

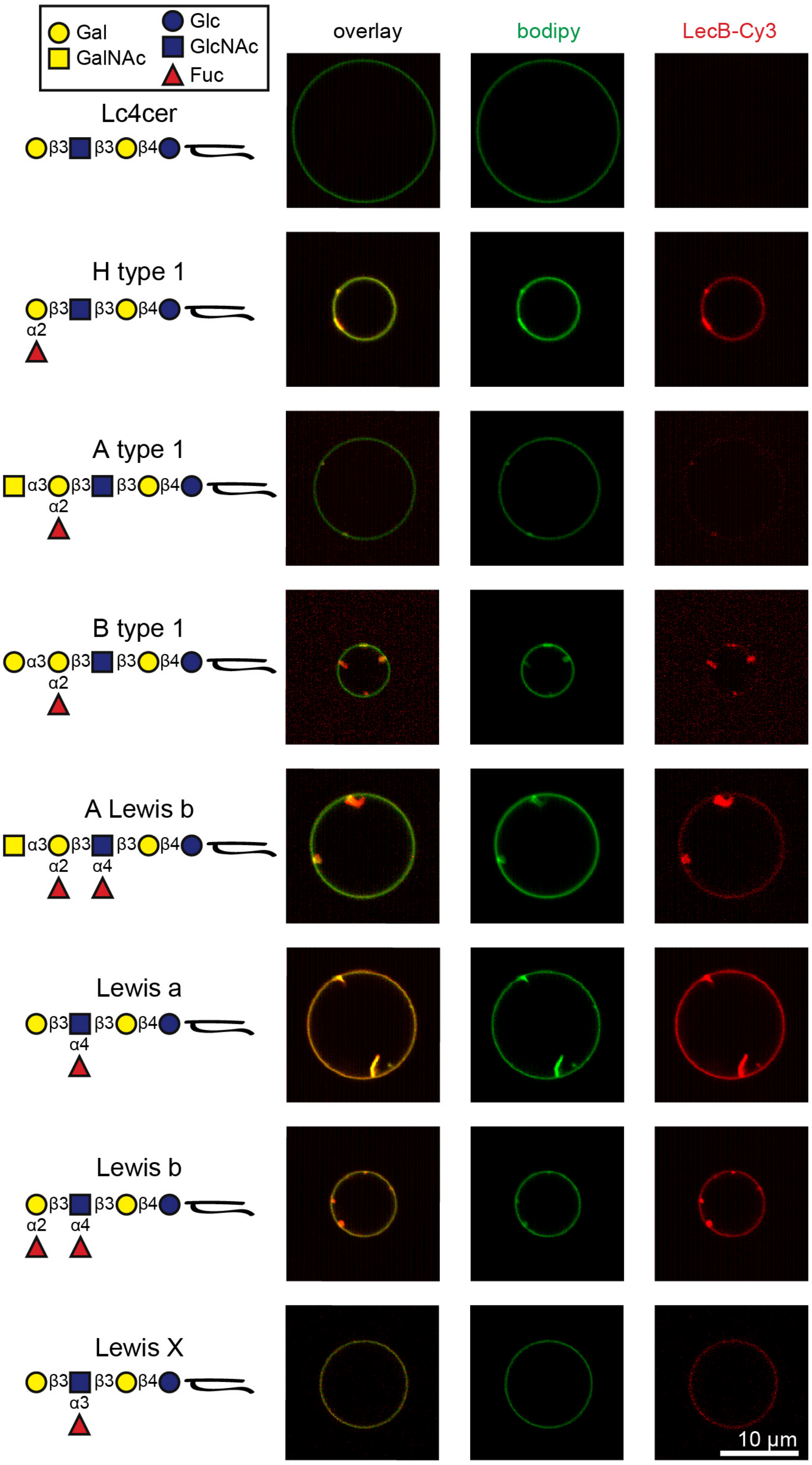

Figure S3: Control experiments related to Fig. 4, part 1

LecB-Cy3 (15 µg/ml, red) was applied to GUVs containing BODIPY-FL-C5-HPC (bodipy; green) as a membrane marker and glycosphingolipids bearing antigens from the type 1 series (lactotetraosylceramide (Lc4cer; non-fucosylated precursor as negative control), H type 1, A type 1, B type 1, A Lewis b, Lewis a, or Lewis b) or the Lewis X antigen from the type 2 series. Schematic structures of the glycolipids are also depicted on the left. Confocal sections along equatorial planes of representative GUVs are displayed.
