# Supplementary material for: The Pseudomonas aeruginosa Lectin LecB Causes Integrin Internalization and Inhibits Epithelial Wound Healing
Source: mBio. 2020 Mar 10;11(2):e03260-19. doi: 10.1128/mBio.03260-19 (PMC7064779; doi:10.1128/mBio.03260-19)
Supplement: FIG S4 [file mBio.03260-19-sf004.pdf]

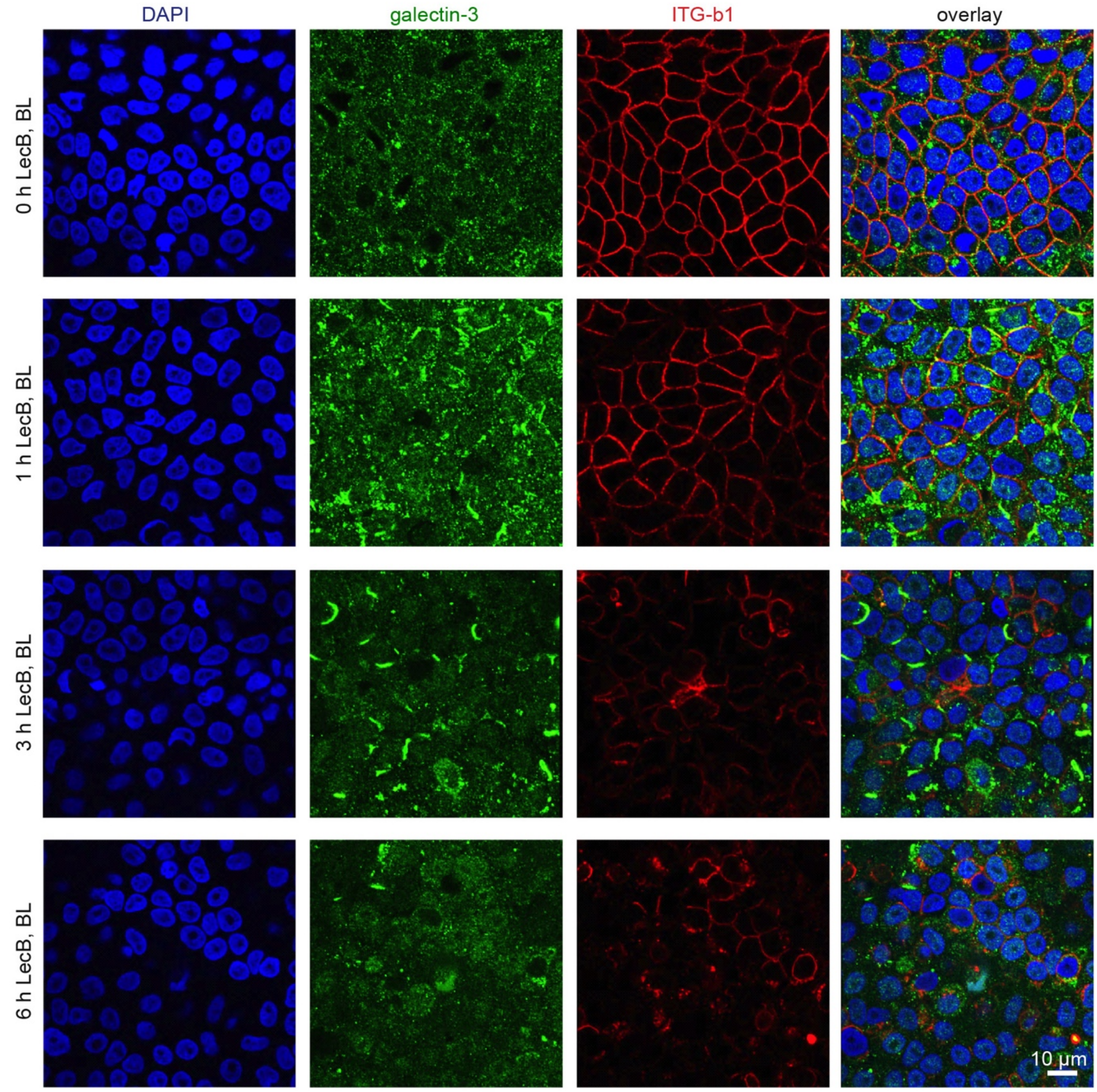

Figure S4: Control experiments related to Fig. 4, part 2

LecB was applied basolaterally to polarized MDCK cells grown on transwell filters. After fixation, endogenous galectin-3 (green), β1-integrin (red), and nuclei (blue) were stained. Representative confocal sections (x-y sections) from a z-level 3 μm above the transwell filter surface are displayed.
