# Supplementary material for: The Pseudomonas aeruginosa Lectin LecB Causes Integrin Internalization and Inhibits Epithelial Wound Healing
Source: mBio. 2020 Mar 10;11(2):e03260-19. doi: 10.1128/mBio.03260-19 (PMC7064779; doi:10.1128/mBio.03260-19)
Supplement: FIG S5 [file mBio.03260-19-sf005.pdf]

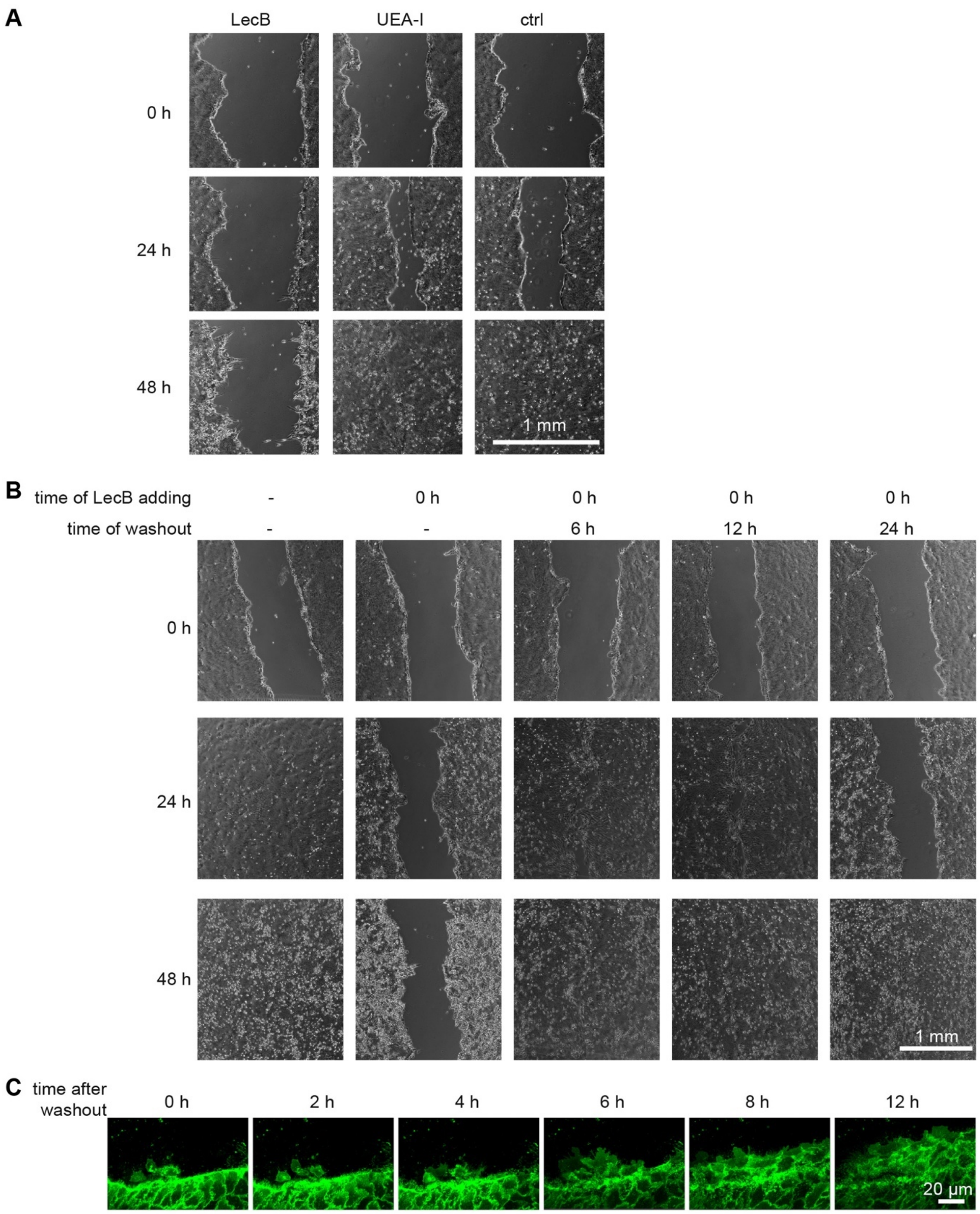

Figure S5: Control experiments related to Fig. 5, part 1

(A) Wound healing assays with monolayers of MDCK cells treated with 50 µg/ml UEA-I or LecB. (B) Wound healing assays with monolayers of MDCK cells in which LecB was washed out again as indicated. (C) Regeneration of cell migration at the wound edge of MDCK cells stably expressing ML-GFP (green) treated for 6 h with LecB and then washed out for the indicated times.
