# Supplementary material for: The Pseudomonas aeruginosa Lectin LecB Causes Integrin Internalization and Inhibits Epithelial Wound Healing
Source: mBio. 2020 Mar 10;11(2):e03260-19. doi: 10.1128/mBio.03260-19 (PMC7064779; doi:10.1128/mBio.03260-19)
Supplement: FIG S6 [file mBio.03260-19-sf006.pdf]

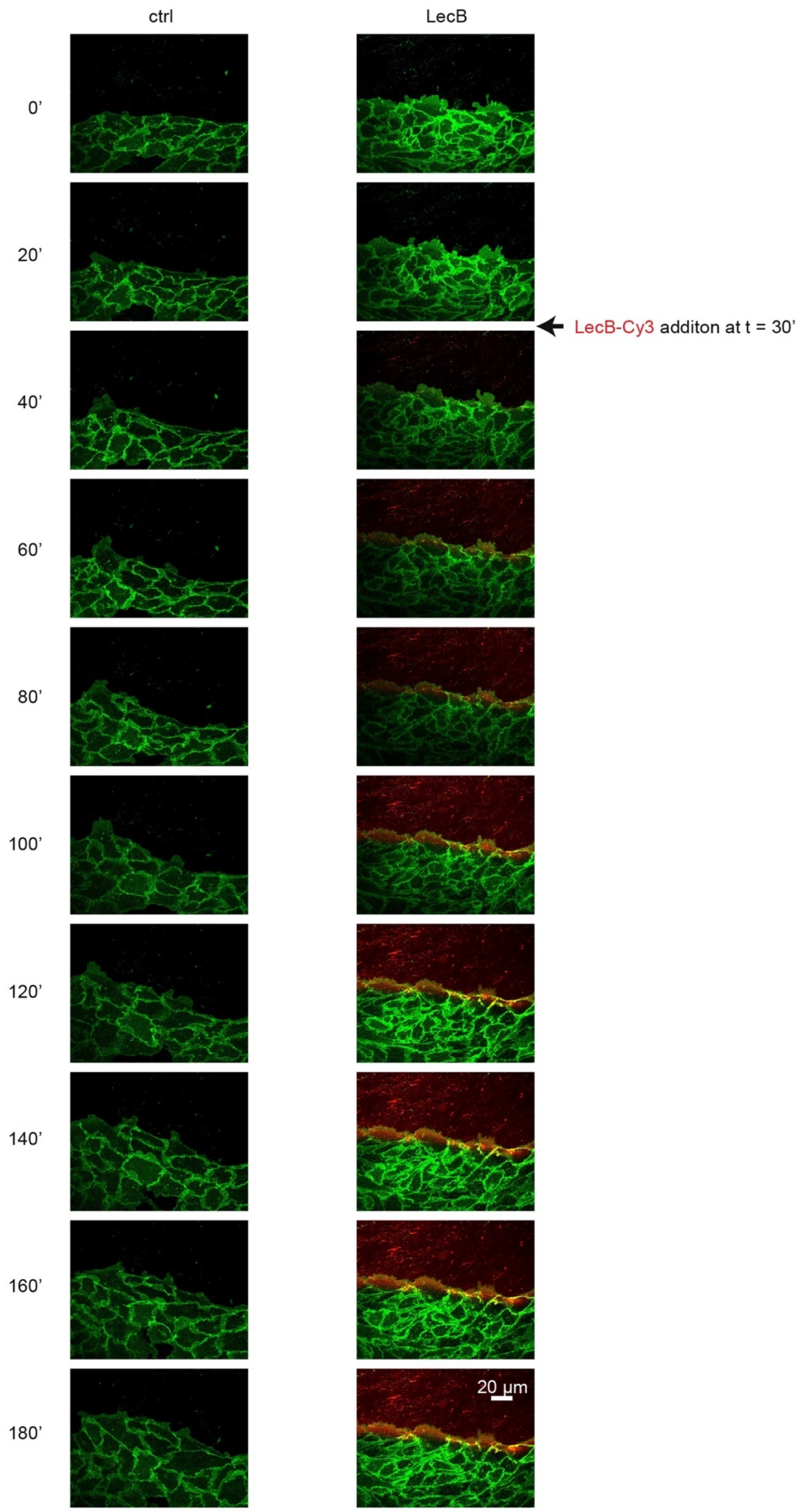

Figure S6: Control experiments related to Fig. 5, part 2

MDCK cells stably expressing ML-GFP (green) were wounded and observed with a confocal microscope. After 30 min, LecB-Cy3 (red) was added to one sample, whereas the other sample was left untreated (ctrl).
