# Supplementary material for: The Pseudomonas aeruginosa Lectin LecB Causes Integrin Internalization and Inhibits Epithelial Wound Healing
Source: mBio. 2020 Mar 10;11(2):e03260-19. doi: 10.1128/mBio.03260-19 (PMC7064779; doi:10.1128/mBio.03260-19)
Supplement: FIG S7 [file mBio.03260-19-sf007.pdf]

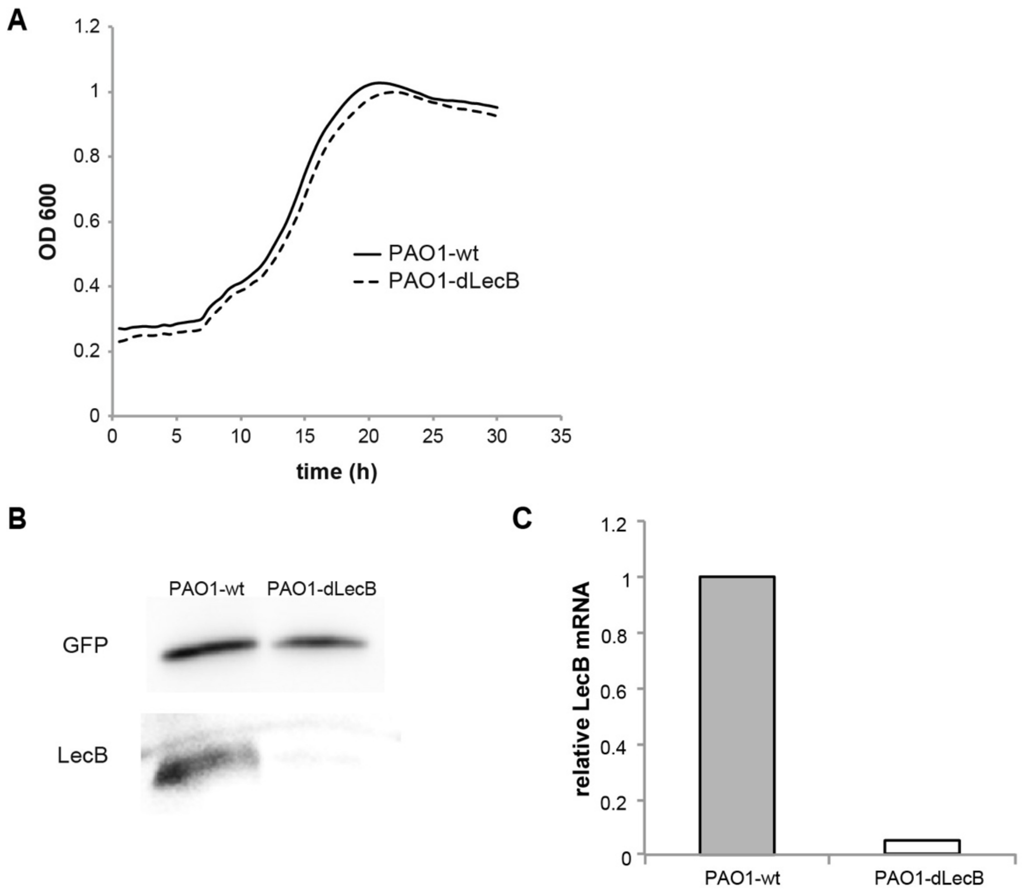

Figure S7: Control experiments related to Fig. 6

(A) OD-600 growth curves from wt PAO1 (PAO1-wt) and LecB-knockout strains (PAO1-dLecB) grown in LB medium at 37 °C. (B) – (C) The presence of LecB was probed in PAO1-wt and PAO1-dLecB, both stably expressing GFP, by WB (B) and semi-quantitative qPCR with rpoD as reference (C).
