# Supplementary material for: The Pseudomonas aeruginosa Lectin LecB Causes Integrin Internalization and Inhibits Epithelial Wound Healing
Source: mBio. 2020 Mar 10;11(2):e03260-19. doi: 10.1128/mBio.03260-19 (PMC7064779; doi:10.1128/mBio.03260-19)
Supplement: TABLE S2 [file mBio.03260-19-st002.pdf]

Table S2: Lists of used primary and secondary antibodies (WB … Western Blot, IF … immunofluorescence, IP … immunoprecipitation, surf. stain … used for surface staining in live cells, milk … milk used as blocking agent, methanol … cell fixation with methanol)

Primary antibodies:

antibody target	generated in species	supplier	product number	dilution (application)
α3-integrin	mouse	BD Biosciences	611044	1:200 (IF, methanol); 1:1000 (WB)
β-actin	mouse	Sigma Aldrich	A5316	1:1000 (WB)
β-catenin	rabbit	Abcam	ab32572	1:250 (IF)
β1-integrin	mouse	Thermo Scientific	MA1-19105	1:200 (IF); 1:1000 (WB, milk)
β1-integrin	goat	R&D Systems	AF1778	1:100 (IF)
β1-integrin	mouse	Millipore	MAB2000	1:200 (IF); 1:1000 (WB, milk); 1:200 (IP)
β1-integrin (4B4)	mouse	Beckman Coulter	6603113	1:100 (surf. stain)
β1-integrin (9EG7)	rat	BD Biosciences	550531	1:100 (surf. stain)
β1-integrin (mAB 13)	rat	BD Biosciences	552828	1:100 (surf. stain)
β1-integrin(AIIB2)	rat	Millipore	MABT409	1:100 (surf. stain)
E-cadherin	mouse	BD Biosciences	610182	1:200 (IF)
laminin (antibody detects β1 and γ1 of LM-511 in MDCK cells (*37*))	rabbit	Sigma Aldrich	L9393	1:100 (IF)
Lamp1	rabbit	Cell Signaling	9091	1:100 (IF)
LecB	rabbit	Eurogentec	newly produced	1:1000 (WB)
Rab9	rabbit	Cell Signaling	5118	1:100 (IF)
ZO-1	rat	Millipore	MABT11	1:50 (IF)

Secondary antibodies:

antibody target	conjugation	supplier	product number	dilution (application)
anti-mouse	HRP	Cell Signaling	7076	1:2000 (WB)
anti-mouse	Alexa488	Thermo Fisher	A21202	1:200 (IF)
anti-mouse	Cy3	Jackson Immunoresearch	715-166-1500	1:200 (IF)
anti-mouse	Alexa647	Thermo Fisher	A21236	1:200 (IF)
anti-rabbit	HRP	Cell Signaling	7074	1:2000 (WB)
anti-rabbit	Alexa488	Thermo Fisher	A21206	1:200 (IF)
anti-rabbit	Cy3	Jackson Immunoresearch	711-166-152	1:200 (IF)
anti-rabbit	Alexa647	Thermo Fisher	A21245	1:200 (IF)
anti-rat	Alexa488	Thermo Fisher	A21208	1:200 (IF)
anti-rat	Alexa647	Thermo Fisher	A21247	1:200 (IF)
anti-goat	Alexa647	Thermo Fisher	A21447	1:200 (IF)
